# Privacy-first health research with federated learning

**DOI:** 10.1038/s41746-021-00489-2

**Published:** 2021-09-07

**Authors:** Adam Sadilek, Luyang Liu, Dung Nguyen, Methun Kamruzzaman, Stylianos Serghiou, Benjamin Rader, Alex Ingerman, Stefan Mellem, Peter Kairouz, Elaine O. Nsoesie, Jamie MacFarlane, Anil Vullikanti, Madhav Marathe, Paul Eastham, John S. Brownstein, Blaise Aguera y. Arcas, Michael D. Howell, John Hernandez

**Affiliations:** 1 Google, Mountain View, CA USA; 2 Biocomplexity Institute, University of Virginia, Charlottesville, VA USA; 3 Department of Computer Science, University of Virginia, Charlottesville, VA USA; 4 Computational Epidemiology Lab, Boston Children’s Hospital, Boston, MA USA; 5 Department of Epidemiology, Boston University, Boston, MA USA; 6 Department of Global Health, Boston University, Boston, MA USA; 7 Harvard Medical School, Boston, MA USA

**Keywords:** Medical research, Mathematics and computing

## Abstract

Privacy protection is paramount in conducting health research. However, studies often rely on data stored in a centralized repository, where analysis is done with full access to the sensitive underlying content. Recent advances in federated learning enable building complex machine-learned models that are trained in a distributed fashion. These techniques facilitate the calculation of research study endpoints such that private data never leaves a given device or healthcare system. We show—on a diverse set of single and multi-site health studies—that federated models can achieve similar accuracy, precision, and generalizability, and lead to the same interpretation as standard centralized statistical models while achieving considerably stronger privacy protections and without significantly raising computational costs. This work is the first to apply modern and general federated learning methods that explicitly incorporate differential privacy to clinical and epidemiological research—across a spectrum of units of federation, model architectures, complexity of learning tasks and diseases. As a result, it enables health research participants to remain in control of their data and still contribute to advancing science—aspects that used to be at odds with each other.

## Introduction

Protecting privacy is crucial in designing, running, and interpreting health studies. However, most health research to date uses data stored in a centralized database (i.e., a database stored in a single site), where analysis and model fitting is done with full access to the sensitive underlying data. Recent advances in distributed machine learning (i.e., machine learning utilizing data stored across two or more sites) enable building complex machine-learned models without necessitating such centralized databases. Federated learning is a subfield of machine learning where multiple participants—sometimes referred to as devices or clients—collaborate in learning a joint model^1^. Federated learning techniques enable calculation of research study endpoints in a privacy-preserving fashion such that private data never leaves a given device (e.g., a research participant’s smartphone, wearable or implanted device) or system (e.g., academic research center, clinical trial site or medical data repository). Each client’s raw data are stored locally and remain under control of and private to that participant. Only focused model updates leave the clients^1^, enabling the aggregation of learned patterns into a single global model without raw data disclosure. The communication between clients can be peer-to-peer but typically involves a central orchestrator that receives and aggregates clients’ updates.

The federated learning approach enables two types of benefits. First, a higher quality model can be learned by leveraging a broader set of data points, beyond what could be done with the data held by any one participant or data silo. This is particularly important for modern machine learning models that often involve large numbers of parameters and by extension require large amounts of data for training. The second benefit is privacy—everyone involved keeps their raw and—in general—sensitive data local and private. Differential privacy is directly incorporated into the approach to protect individuals’ privacy.

These characteristics make federated learning particularly appealing for scalable health research, where a large fraction of the population may want to contribute to novel health findings, but have reservations about sharing raw data and digital signals. While federated learning has generated significant interest in the machine learning community in recent years, with a specific focus on smartphone-based analytics and learning^2^ and learning across data silos of various healthcare systems^3–7^, its applications to clinical and epidemiological studies over individuals’ data are only beginning to emerge—for example in a new study on respiratory infections^8^. At this point, however, only specific large homogenous units of federation, such as at the level of a healthcare system, have been studied in detail in prior work, and the focus has been on traditional classification tasks.

As a result, considerable challenges and open questions remain that to our knowledge have not been systematically studied to date. In particular, health research often involves a relatively small number of participants (small N) in each study, limited number of “rows” of data per participant, a large number of multifactorial variables, and potentially unequal levels of patient participation. Specifically, health study data is typically non-IID—not independent and identically distributed—which is compounded by the fact that in the federated regime, individual data points are distributed across many devices that participate asynchronously. Since many machine learning methods work under the assumption of IID, it is important to empirically examine its effects in a federated setting as well. Further, in a large number of clinical studies, the focus is not on prediction, but correlational analysis to understand the associations between different factors, and hypothesis testing. Prior methods are often ad-hoc, which can be a problem in generalizing to a new dataset with a potentially different level of federation. Here we examine the broader spectrum of units of federation—from the extreme of each subject being one unit to large units on a per-country basis—and a spectrum of machine learning tasks and complexities. Finally, prior studies have not fully considered privacy, which is not guaranteed by default in an arbitrary federated learning setup, and needs to be treated, implemented, and studied explicitly.

Our work demonstrates the successful use of federated learning in the presence of these challenges in homogeneous data silo settings (i.e., where the output of federated computation from one data silo is composable with the output from another silo). Specifically, in this work we reproduce eight diverse health studies in terms of study design, statistical analysis and sample size, spanning the past several decades in a purely federated setting, where each unit of federation keeps their data private but still contributes to the aggregate model. We randomly sampled seven observational studies and one clinical trial that generated new knowledge on various clinical and epidemiological problems, and made the underlying raw data publicly available. The focus of these studies ranges from diabetes to heart disease to SARS-CoV-2 and MERS-CoV to patient mortality prediction based on electronic medical records.

The complexity of the models and underlying data also cover a wide gamut: from regression models with a few variables and relatively small number of patients to deep neural net models with 17,527,793 parameters, involving tens of thousands of patients. We employed such powerful models to incorporate complex and/or unstructured signals, such as free form text from caregiver notes within electronic medical records, and sequential data (e.g., time series of hospital encounters where each encounter has its own complex structure). We note that recent advances in federated learning on images point the way towards including further multi-modal signals in distributed models; our work focuses on textual, categorical, and sequential data.

Finally, to test various units of federation, we experiment with the extreme case of each patient being its own unit, as well as with groups of patients. Four out of the eight datasets are studied at *both* the individual level of federation as well as larger generally non-IID units (silos), such as hospital unit, community, or country. These groupings were taken from the original data structure to mimic real-world settings and complexities as closely as possible and are summarized in Table 1. To explore a broader range of types of silos when the original data does not contain such naturally-occurring silos, we also silo the data randomly using a Dirichlet distribution and run cross-silo experiments on the resulting grouping. *Additionally, rather than developing a custom technique to federate learning of one specific class of models as done in prior work, we demonstrate how such encompassing work can be achieved within the unified framework of TensorFlow*.

**Table 1:** Summary of datasets and methods reproduced in this work.

Study Topic	Manuscript	Study Design	Unit of Analysis	*N*	Statistical Model	Additional Methods	Measure	Covariates
Heart Failure	Chicco & Jurman^11^	Cohort Study	Individual	299	Logistic Regression	N/A	AUC	12
Diabetes	Smith, et al.^27^	Cohort	Individual	768	Neural network with 1 hidden layer	N/A	AUC	8
MIMIC-III	Johnson et al.^12^	Database	Individual & Dirichlet grouping	53,423	Deep neural network	N/A	N/A	92
SARS-CoV-2	Rugge et al.^13^	Cohort Study	Individual & random size grouping	9275	Logistic Regression	N/A	Odds Ratio	3
Avian Influenza	Fiebig et al. ^28^	Case Series	Individual & country grouping	294	Logistic Regression	Forward/Backward Selection	Odds Ratio	4
Bacteraemia	Harris et al.^29^	Case Control	Individual	159	Logistic Regression	Forward/Backward Selection	Odds Ratio	12
Azithromycin	Oldenburg et al.^30^	Cluster Randomized Trial	Individual & community of residence grouping	1712	GLM with a Binomial response and the log link	Standard Errors Clustered	Risk Ratio	0
Tuberculosis	Ohene et al.^31^	Case Series	Individual & multi-center grouping	3342	Logistic Regression	N/A	Odds Ratio	3

*GLM* Generalized Linear Model, *AUC* Area under the Receiver Operating Characteristic Curve.

## Results

### Overview

We identified eight studies with publicly available data. These represented a gamut of study designs (four cohort studies, three case series, one clinical trial), statistical tasks (three prediction tasks in terms of AUROC, five inference tasks in terms of relative risks) and sample sizes (Range, 159–53,423) (Table 1). In all studies, we compare—side by side—the results of the originally published model with its federated counterpart, and with/without central and local differential privacy. The comparison is done across several key dimensions: in terms of robustness of the model—how well does it generalize and capture unseen data; model interpretation—are optimal model parameters found in all cases and do they have the same values; and finally scalability—can federated learning support studies with a wide range of the number of participants and the amount of data each subject generates (both small and large), and the impact of privacy constraints. We find that the results from federated learning are on par with centralized models, both in terms of performance (measured as Area Under the Receiver Operating Characteristic Curve) and interpretation (measured as odds ratio or coefficient) (Table 2). Unlike prior work, which is typically tailored to a specific fixed setting, we use TensorFlow for all our analyses, which provides an openly available, well-documented systematic and unified methodology for federated learning.

**Table 2:** Summary of original and federated results reproduced in this work.

Study Topic	Sample Results	Comparison Metric	Traditional Centralized Model^a^	Federated Replications	
				Per-Patient	Per-Silo^b^
Heart Failure	1. Survival Prediction (full model) 2. Survival Prediction (with variable selection)	AUC	0.82 0.82	0.85 0.83	N/A
Diabetes	1. Diabetes prediction at 5-years	AUC	0.84	0.875	N/A
MIMIC-III	1. Inpatient mortality prediction	AUC	0.780± 0.012	0.777 ± 0.011	0.777 ± 0.014
SARS-CoV-2	1. CV2+ve in Female vs. Male 2. CV2+ve in Recent vs. Never Cancer	OR	0.35 (0.32–0.38) 1.88 (1.36–2.60)	0.35 (0.32–0.38) 1.99 (1.45–2.68)	0.35 (0.32–0.38) 2.07 (1.50–2.86)
Avian Influenza	1. Fatality with each day before hospitalization 2. Fatality in Indonesia vs. group of countries	OR	1.33 (1.11–1.60) 0.23 (0.04–1.27)	1.34 (1.12–1.61) 0.25 (0.05–1.37)	1.33 (1.11–1.60) 0.24 (0.04–1.33)
Bacteraemia	1. Relapse with line-associated infection source 2. Relapse with presence of immunosuppression	Coefficient	1.57 (SE: 0.45) 1.07 (SE: 0.41)	1.59 (SE: 0.23) 1.12 (SE: 0.30)	N/A
Azithromycin	1. Adverse events in azithromycin treated	Coefficient	−0.11 (SE: 0.09)	−0.29 (SE: 0.19)	N/A
Tuberculosis	1. Extrapulmonary TB in individuals with HIV	Coefficient	1.16 (SE: 0.09)	1.35 (SE: 0.08)	0.15 (SE: 0.07)^c^

Odds ratios shown as point estimates (95% confidence intervals). Model beta coefficients shown as estimate (standard error).

*OR* odds ratio, *AUC* Area under the Receiver Operating Characteristic Curve.

^a^As reported in original study or replicated in centralized fashion with statsmodel.

^b^Example silos include hospital level, patient groups, and country level. Not all existing datasets allow meaningful grouping at various levels.

^c^Problem under-specification issue—see additional details in Supplementary Discussion 2 (extrapulmonary tuberculosis).

Furthermore, we contrast privacy properties and the utility of these new distributed methods with traditional central differential privacy methods (see “Methods”) used in classical settings. As there are growing concerns about the ability to maintain the privacy of research participant data as it becomes increasingly feasible to re-identify individuals through combining multiple sources of electronic health data^9,10^ we show that new methods involving federated learning and differential privacy can provide very strong privacy protections with minimal reduction in utility.

This work’s primary focus is on cross-device (cross-patient) settings, where the unit of federation is a single individual. However, we also show the same approach generalizes to the cross-silo setting, where the unit of federation is larger, such as a hospital unit, a healthcare system, or even a country (see Supplementary Note 1 for a formal problem definition). To do so, we concentrate on two broad classes of models used in medical research: logistic regression (LR) and deep neural network (DNN) (Supplementary Discussion 1). While logistic regression is a special instance of a broader class of neural models, we treat it separately as it still underpins a large fraction of health studies done to date, due to its relative simplicity and interpretability. To quantify differences in performance and interpretation of models trained in a centralized fashion to those trained in a distributed way, we use the same mathematical formulation of the core model (e.g., model formula, loss function) and apply it to the same data. The key difference is in how the training data is stored and accessed (centralized vs. federated) and how the model optimization is implemented.

**Fig. 1:**
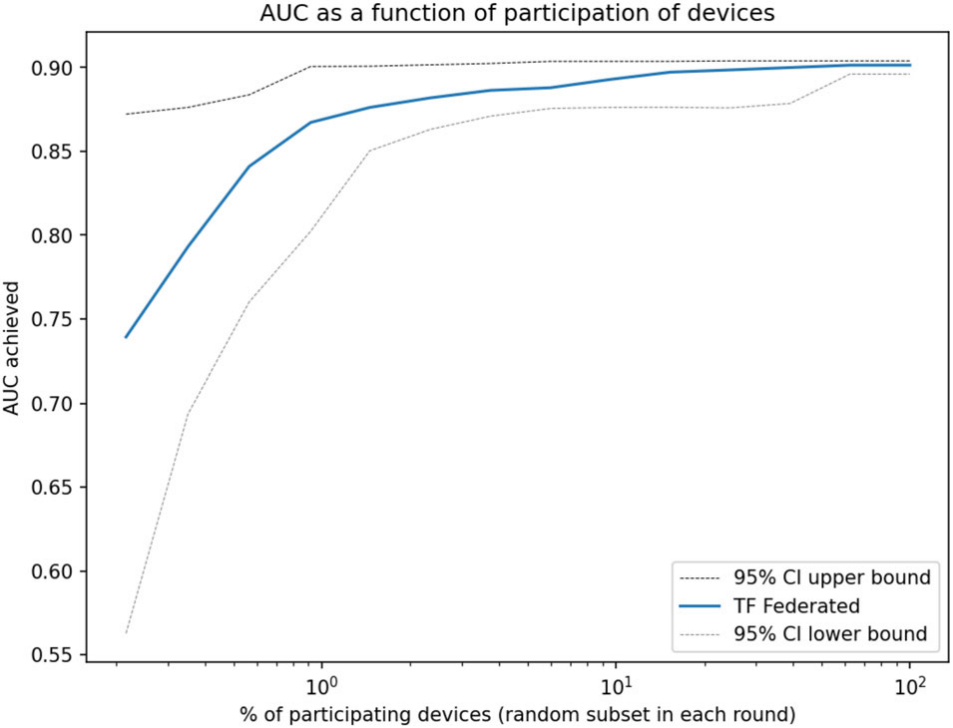
Area under the ROC curve (AUC) as a function of fraction of participants in each federated (server) round of learning for replicated model of SARS-CoV-2 and Cancer. Shown in log scale to highlight details at the low participation levels. We see that even at a 2% participation rate, the model still achieves 99% of the maximum attainable AUC. We observe this pattern across all the datasets studied. 80% of the whole dataset was used to train the model and the rest 20% used for validation.

Since in general in the federated setting not all participants may be available at any one time, we explore model quality as a function of subjects’ participation rate. Across the datasets, we find that only a minority of clients need to participate in any one round of federated learning to achieve the maximum attainable performance in terms of AUC (Fig. 1). These sub-populations are sampled at random with replacement for each round. Based on our analyses, we expect that 2% of the randomized participants would suffice to obtain 99% of the expected model performance in terms of AUC. This makes the federated setup quite robust to platform-independent bias caused by device dropout, as only a small proportion of the total devices need to be available at any one time (Supplementary Discussion 3). Furthermore, all experiments in this work were performed on an inexpensive commodity desktop computer and took only an order of minutes of runtime until convergence. As we discuss below, while federated learning introduces a communication overhead as participating devices need to communicate, parts of the computation are distributed across devices, which results in computational requirements similar to the ones needed in a traditional (i.e., centralized) approach.

We now turn to briefly describe all datasets used in this study (Table 1) along with prior work we reproduce here in a federated setting. The datasets and models reproduced here vary along many axes, namely the number of examples, class balance/imbalance, number of independent variables, the amount and nature of the signals leveraged (e.g., continuous, discrete, categorical, textual, embedded, time series), focus on various metrics (e.g., ROC AUC, hypothesis testing, odds ratio, coefficient interpretation, test of statistical significance), and model architectures (e.g., regression models, neural networks of various depth, sequential models). We discuss general challenges in reproducing statistical models in Supplementary 5. In this section, we focus on a sample of diverse results and report the remainder in Supplementary Discussion 2. We highlight that in all datasets tested and across all axes considered, the federated method reaches the same conclusion as the original work.

### Heart failure

The Heart Failure Clinical Records Dataset from the University of California Irvine data repository involves 299 individuals with left ventricular systolic dysfunction and New York Heart Association class III or class IV heart failure ranging from 40 to 95 years of age^11^. The dataset was collected in 2015 from the Faisalabad Institute of Cardiology and the Allied Hospital in Faisalabad in Pakistan. The dataset is used to predict survival, based on 13 attributes including age, sex, blood pressure, left ventricular ejection fraction, diabetes, anemia, and creatinine levels.

The original work presents two logistic regression models—one with all variables and one with only three observed variables (ejection fraction, serum creatinine, and time of followup in months). Our federated setting achieves 0.85 AUC (95% confidence interval of 0.85–0.86) in the full model formulation (cf. 0.82 in the original work) and 0.83 AUC (0.82–0.84) in the latter setup with variable selection (cf. 0.82 in the original work). The higher AUC score in our setting is due to the addition of regularization while optimizing model parameters, which also allows the new method to subsume the semi-manual variable selection done in the original work. Mirroring the original study, all metrics are reported as means over 100 executions with randomized training-testing data splits. Adding a central differential privacy module (Supplementary Discussion 4) reduces AUC to 0.83 (0.82–0.84) for the full model (cf. 0.82 in the original work which does not consider any DP protections), but provides strong guarantees (*ε* = 0.165 and *δ* = 10^−5^). With local DP, the federated architecture achieves also 0.83 AUC (0.82–0.84) with local *ε* = 1.36 and local *δ* = 10^−9^ per round. We note this is a very small dataset containing only 299 examples and this experiment demonstrates our methods apply also in situations where data is limited.

### Electronic medical records (MIMIC-III)

MIMIC-III is a freely available critical care electronic health records (EHR) database involving comprehensive data from ~40,000 distinct patients age 16 and older, spanning over 53,000 hospital admissions to Beth Israel Deaconess Medical Center between 2001 and 2012^12^. The dataset contains 4579 charted observations and 380 laboratory measurements associated with hospital admissions. Each patient in the dataset has a time series of medical encounters involving procedures, medications, diagnoses and other complex signals, such as medical notes. Compared with other tasks with only numerical and simple categorical features, these medical notes are much more complex and require more sophisticated natural language processing models to interpret. In this work, we developed a deep neural network that takes medical note tokens into large embedding layers, and learns each token’s representation end-to-end alongside the attached fully connected layers. We further extend this to work in a federated learning environment. This allows us to test federated learning in a setting where each patient is represented by a large amount of diverse and multi-modal data points on a timeline.

We build a deep neural network to predict inpatient mortality with data up to 24 h after admission, using patient age, gender, Clinical Classification Software diagnosis codes, RxNorm medication codes, Current Procedural Terminology procedure codes, and free-text medical notes as input variables. The model architecture contains an input layer, three hidden layers with 512, 256, and 128 neurons respectively, and an output layer with a sigmoid activation function (Supplementary Discussion 1, Supplementary Fig. 2). We train the model using the Adam optimizer with a learning rate of 0.01. In addition, and use L1 regularization with magnitude 0.0001 and L2 with 0.01.

To explore different levels of federation, we partition the dataset on a per-patient basis (unit of federation is a single patient) and in groups of patients (per-silo basis). In particular, the per-patient federation follows the cross-device federated learning setting, where each client holds data of a single patient, while the per-silo federation setting splits patients into multiple groups (silos) using a Dirichlet distribution, which simulates the case each hospital or organization holds their patients’ data.

To demonstrate the efficacy of federated learning on this dataset, we compare the ROC curve of three different experiments: (1) TF centralized model: A traditional server-side trained model assumes all data is available on a centralized server. (2) TF federated cross-device model: A model trained on clients on a per-patient basis. Each training round has 16 participating patients, and we trained the model for 500 rounds. (3) TF federated cross-silo model: A model training on clients on a per-silo basis. We use a Dirichlet distribution with parameter alpha of 10 to randomly group all patients to 20 groups of various sizes according to the distribution, and select 5 groups at random to participate in each federated training round. The median and interquartile range of patient counts in each group are 200 and 40, respectively.

**Fig. 2:**
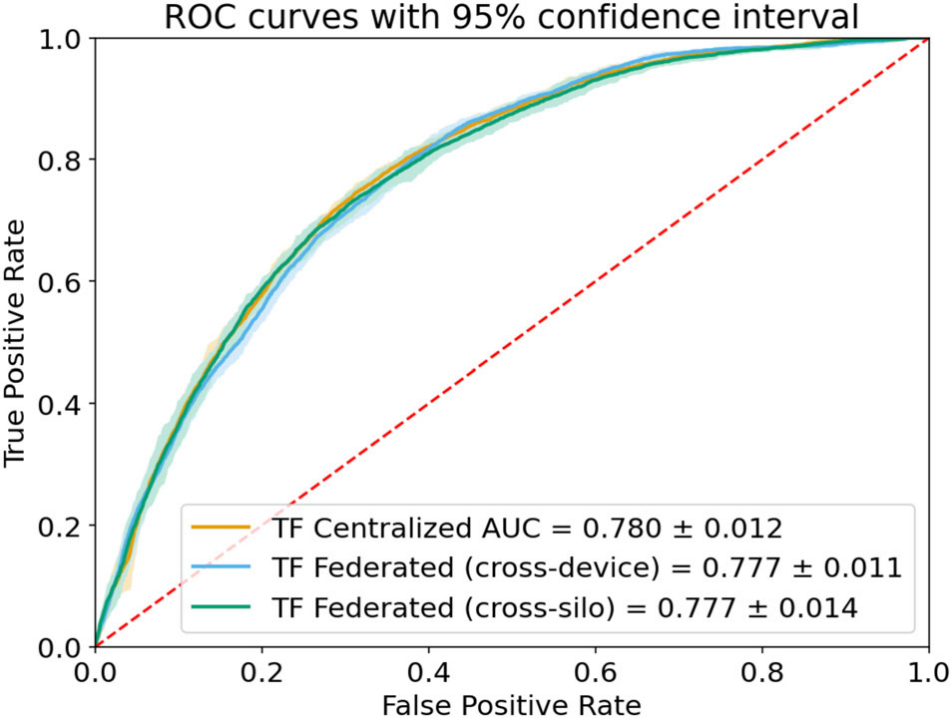
Receiver operating characteristic curves for the three learning setups on MIMIC-III data predicting inpatient mortality. Shaded areas show 95% confidence intervals.

We measure the performance of three models using the AUC metric and find that all three models achieve comparable performance with greatly overlapping confidence intervals (Fig. 2).

### SARS-CoV-2 and cancer

The Malignancy in SARS-CoV-2 Infection database is a large community-based registry of over 84,000 people who were tested between February 22 and April 1, 2020 for SARS-CoV-2 in the Veneto region of Italy^13^. The dataset has been used to understand the risk of SARS-CoV-2 infection and health outcomes, based on age, sex, and cancer history.

Rugge et al. (2020) presented the following observations:

1. The risk of hospitalization is lower among females (OR, 0.35; 95% CI, 0.32–0.38).
2. Compared to young people, COVID-19 positive patients aged 70 years or more were at a higher risk of hospitalization (OR, 4.02; 95% CI, 3.58–4.52).
3. Individuals diagnosed with cancer within 2 years before acquiring the infection showed the highest risk of hospitalization (OR, 1.88; 95% CI, 1.36–2.60).

**Fig. 3:**
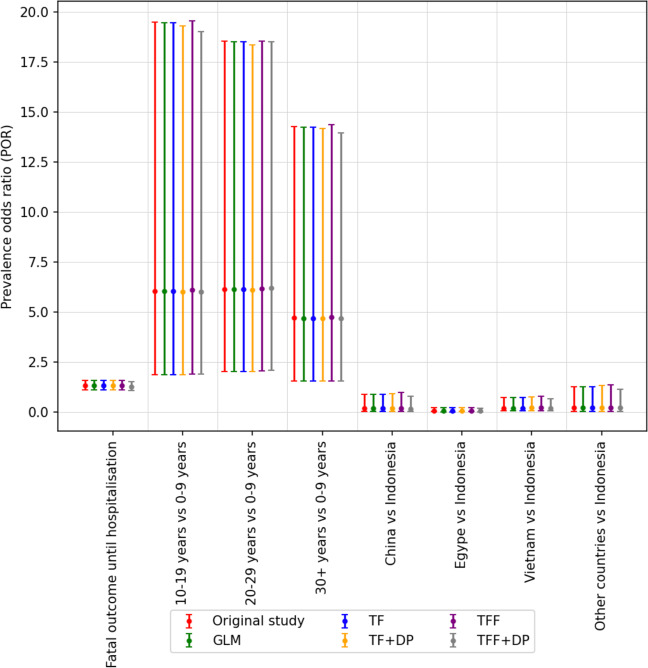
The odds ratio other than red color are generated using our models. The odds ratio generated by our models are consistent with the odds ratio of the original study. The vertical bar along with each coefficient shows 95% confidence level of corresponding ratio.

**Fig. 4:**
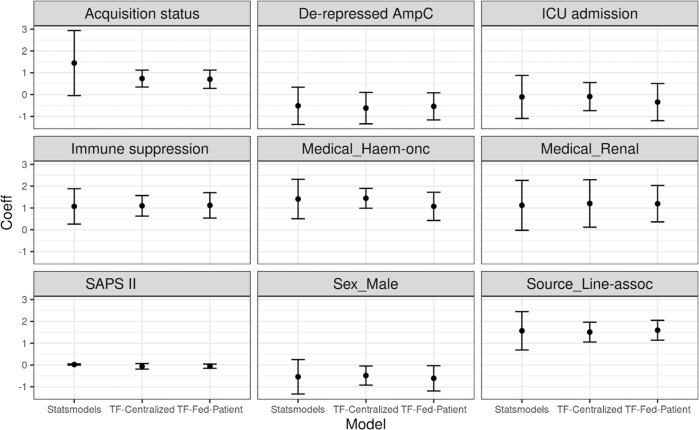
The estimated coefficients of Statsmodels (GLM), TF-Centralized (Tensorflow Probability) and TF-Fed-Patient (Tensorflow Probability with Federated Learning, using patient as the unit). The plots show the coefficients and their 95 %confidence intervals of nine variables of different univariate logistic regression models. The significance of all models and variables is almost consistent with the original study: eight over nine variables have the same conclusions and only one (Acquisition status) does not (TF-Centralized and TF-Fed-Patient both show it is significant, while GLM and the original study state otherwise). In the original study, the variable has a *p* value of 0.06 which lies near the borderline of significance (*p* ≤ 0.05).

We split our experiments into six sections and compare performance between both centralized and federated learning models. To test various units of federation, we experiment with the extreme case of each patient being its own unit (Supplementary Figs. 3 and 4), as well as with groups of patients (Supplementary Fig. 5). Supplementary Fig. 3 shows the ability of the federated approach to learn coefficients equivalent with the original work. Supplementary Fig. 4 shows an agreement in odds ratios across the models. The full remainder of the experiments are reported in Supplementary Discussion 2 and key results summarized in Table 2, Figs. 3 and 4. Each of our models reproduces the results of the original study.

## Discussion

This paper focuses on federated learning across individual patients’ data that can be stored independently of each other. By contrast, most existing applications of federated learning to health research involve several bulk data holders (for example, academic research centers, pharmaceutical companies, or hospitals) collaboratively training models on their entire joint datasets, containing data about many individuals, all at once^14^. The two approaches are termed “cross-device” and “cross-silo” federated learning respectively, and are described in-depth in Kairouz et al.^1^.

Cross-silo federated learning has already been applied in the healthcare arena to power clinical research among participating hospitals or pharmaceutical companies^15,16^. In these applications, each participant holds a significant amount of data, sufficient for independent analysis; federated learning improves the quality of this analysis by leveraging data held by multiple participants. By contrast, in this work, we focus on those scenarios commonly found in epidemiological health studies, specifically studies with many participants, each of whom has relatively small amounts of non-IID, labeled data. The approach described here can be appropriate for health studies involving smartphone/wearable data and virtual clinical studies (also called decentralized clinical studies) that directly recruit individual research participants without relying on clinical sites for recruitment.

Applications of cross-device federated learning for medical research include: (1) training models on data that is held directly by individuals—for example, health or behavioral data collected on their phones—without requiring a trusted centralized collector, and (2) making use of data signals that are too sensitive or resource-intensive to transmit to a central location. There exists significant prior work evaluating federated learning in the cross-device setting, where many clients each hold their own training examples^17–19^. Especially when combined with differential privacy, the literature demonstrates privacy gains in these scenarios^14,20^.

While this paper focuses on model training via federated learning (FL), federated *analytics* (FA)—the application of data science techniques to data that is stored locally on client devices^21^—holds similar promise for health research. Within the scope of federated analytics lie averages, histograms, heavy-hitter identification, quantiles, set cardinality, covariance matrix estimation, clustering, dimensionality reduction, graph connectivity, and more.

In FL as discussed in this paper, the fundamental training procedure is the same no matter the model, supporting the generality of the experimental results, but FA algorithms vary widely. As a result, comparisons between FA algorithms and their classical, centralized counterparts do not necessarily generalize. Some state-of-the-art FA algorithms are highly interactive, like FL, with individual clients able to contribute many times to iteratively refine the results^1^, while others, like federated averaging, can be completed in a single, trivial pass over the clients. For the former, the non-IID nature of the federated data can be significant; for the latter, imperfect client sampling is the only source of divergence from the centralized computation.

Indeed, the major common thread among FA algorithms as compared to their central counterparts is the effect of client sampling on the results. However, because sampling effects depend entirely on the reliability and availability of clients, and these in turn depend on the implementation details of the federated system, we do not attempt to characterize their impact here. Doing so in a general way is an area for further research.

This work demonstrates for the first time that in a broad portfolio of health studies featuring varying study designs, prediction and inference tasks, prediction model types and sizes, levels of federation (individual, hospital unit, community, country) and signal modalities of varying complexities, that models learned in a decentralized privacy-first fashion using federated learning achieve comparable results to the traditional, centrally-trained models. Furthermore, we show that the clinical insights gained from each model are equivalent across these two regimes and that these results hold even when local and central differential privacy protections are added, which is typically not captured in prior work.

Additionally, rather than developing a custom technique to federate learning of one specific class of models as done in prior work, we demonstrate how such encompassing work can be achieved within the unified framework of TensorFlow. A broad range of models can be implemented in this framework, including generalized linear models, risk prediction models, deep neural models, sequence models, and time-to-event models^22^. The methodology introduced here is quite general as it captures a spectrum of units of federation (individual patients/subjects → hospital units → healthcare systems → … → countries) and uses multiple model architectures.By contrast, prior work heavily focused on a single point on this spectrum—learning across silos at the level of healthcare systems and often for a fixed model architecture without differential privacy.

As a result, this work is the first to apply modern and general federated learning methods to clinical studies and demonstrates how research can be done with significantly stronger privacy protection guarantees and without reducing its power or validity. Finally, we find that application of federated techniques to modeling health data introduces new open questions and challenges in terms of a more complex computational framework, limits on arbitrary data exploration, training requirements for analysis and the introduction of platform-dependent bias. These issues require careful consideration at the experimental design stage and are further discussed in Supplementary Discussion 3.

The principal class of limitations stems from the distributed nature of the federated study setup. This by design constraints arbitrary data exploration because such ability would erode the privacy guarantees. We mitigate this by leveraging federated analytics to securely monitor relevant statistics about the pool of participants. For example, distributions over data can be computed in real-time in order to detect possible emerging biases. We elaborate on these points in Supplementary Discussion 3.

Additionally, this work simulates and evaluates applications of federated learning within existing datasets. As such, even though it has provided valuable insights and a confirmation of the validity of its estimates, future prospective studies are needed to reinforce our findings. To that end, we are currently running one such distributed study^8^.

Finally, further work will focus on FL over multimodal datasets involving images, audio, and video. We are also planning to extend—to FL scenarios—interesting prior work on representation learning over sequential EHR data similar to MIMIC-III data studied here.

## Methods

### Overview

A typical health study records each participant or patient as a row of data values. These represent outcomes of measurements on the subject, demographic variables, and other data fields the study tracks. The row also contains an outcome (dependent) variable the study is aiming to explain in terms of the other data fields. The vast majority of studies to date have been run in a “classical” fashion, where such rows of data—each for one subject—are concatenated together and stored in a centralized database table or a spreadsheet accessible to the researchers. Here, we explore an alternative setup where the rows are *not* concatenated, but instead remain decentralized, simulating a setting where the data is generated or stored on the subjects’ devices such as smartphones or wearables. Using federated learning, these private rows contribute to learning the global salient associations between the independent and dependent variables just like in the centralized setting, while keeping the raw and potentially very sensitive data local and under control of each individual participant.

The regime just described sets the unit of federation at a very fine-grained level of individual subjects. As we will see, the approach presented here generalizes without modifications to cover the entire spectrum of federation units: from subject-level single rows, to multiple rows per subject, all the way to patients grouped at a healthcare system level.

To make use of the existing datasets but lift them to a federated setting, we partition the original centralized dataset to simulate the data being physically distributed across research participants, each of which is treated as an individual client and contributing with various participation rates to jointly learn a model. That is merely an artifact of available data for prior studies we reproduce here. With the exception of MIMIC-III, the existing datasets have already been collapsed to one row of data per participant. However, this approach works more generally in a setting where each participant captures multiple data examples, and the aggregation happens as part of the local computation. In that setting, each participant may contribute multiple data rows to the computation, loosening the constraints that early aggregation imposes. This is shown in our experiments on electronic health records, which consist of complex sequential data spanning a period of hospitalization (see Supplementary Discussion 2).

### Privacy technologies

Protecting the privacy of epidemiological study participants is a key motivation of our work. Because privacy is not a binary or scalar quality, reasoning about the privacy properties of any system requires a careful evaluation of its threat model, broken down by the actors/participants. A thorough treatment of the privacy threat model for federated learning and related technologies is given in Kairouz et al.^1^. Here, we concentrate our discussion on three core technologies and their compositions: federated learning, secure aggregation, and differential privacy (Supplementary Discussion 4).

### Federated learning

In a Federated learning setting, the data held by clients can only be accessed by the clients themselves. A global computation may involve many clients participating; however, each client keeps its data local, performs local computations over it, and only allows a focused update or summary of what has been computed to be shared with the central orchestrator. The use of focused updates embodies the principle of data minimization: the updates that leave the client are maximally focused on the task at hand, as opposed to the raw data which can be used for a variety of different tasks if it were shared directly. The updates provided by clients only need to be ephemerally held by the recipient server until aggregation can be performed.

The baseline federated learning setting offers a number of practical privacy improvements over centralizing all the training data, but there is currently no formal guarantee of privacy in the baseline federated learning model. Attacks focusing on reversing training data from the updates have been described in the literature^23^. Additionally, the issue of model data memorization may manifest itself in the process of federated learning^24^, just as it does with traditional, centralized machine learning^25,26^. Where it is important to address these concerns, additional privacy technologies such as differential privacy and secure aggregation (see Supplementary Discussion 4) may be used together with federated learning.

### Reporting summary

Further information on research design is available in the Nature Research Reporting Summary linked to this article.

## Supplementary information

Supplementary Information

Reporting Summary

## Data Availability

All data sources used in this manuscript are publicly available. Links for each dataset are available in Supplementary Data.

## Code availability

Code for the analysis is available at https://github.com/google/federated-ml-health.
