# Supplementary material for: Privacy-first health research with federated learning
Source: NPJ Digit Med. 2021 Sep 7;4:132. doi: 10.1038/s41746-021-00489-2 (PMC8423792; doi:10.1038/s41746-021-00489-2)
Supplement: Supplementary file 1 — Supplementary Information [file 41746_2021_489_MOESM1_ESM.pdf]

Privacy-first health research with federated learning

Supplementary Information

Adam Sadilek[1]*, Luyang Liu[1], Dung Nguyen[2,3], Methun Kamruzzaman[2], Stylianos Serghiou[1], Benjamin Rader[4,5], Alex Ingerman[1], Stefan Mellem[1], Peter Kairouz[1], Elaine O. Nsoesie[6], Jamie MacFarlane[1], Anil Vullikanti[2,3], Madhav Marathe[2,3] , Paul Eastham[1], John S. Brownstein[4,7], Blaise Aguera y Arcas[1], Michael D. Howell[1], John Hernandez[1]*

Affiliations:

[1]Google, Mountain View, CA

[2]Biocomplexity Institute, University of Virginia, Charlottesville, VA

[3]Department of Computer Science, University of Virginia, Charlottesville, VA

[4]Computational Epidemiology Lab, Boston Children's Hospital, Boston, MA

[5]Department of Epidemiology, Boston University, Boston, MA

[6]Department of Global Health, Boston University, Boston, MA

[7]Harvard Medical School, Boston, MA

*Corresponding authors: Adam Sadilek (adsa@google.com) and John Hernandez (johnbhernandez@google.com)

Supplementary Note 1

Problem Specification

We are given n clients a_1, a_2, ..., a_n in which each client a_i has in its own control local data D_i. There is a central coordinator C (the server). Our goal is to design a learning algorithm A that serves as a gradient-based learning algorithm to produce a machine learning model across all participating clients[4]. The clients only send (differentially private) gradients back to the central coordinator. The method requires that D_i not be revealed to C.

Supplementary Note 2

Convergence, scalability, and participation

In this work we benchmark more precise but computationally expensive and centralized methods (such as linear solvers, Newton's method, etc.) with more general approaches that scale to larger datasets and can be readily run in a distributed fashion but may be approximate (e.g., stochastic gradient descent -- SGD). We find the best performance with SDG optimizer on the client side and Nadam optimizer for server gradient averaging.[5] The latter leverages gradient update momentum, together with binary cross-entropy loss function. We note a general challenge in stochastic statistical modeling, namely stopping criteria for the learning process.

This is not specific to federated setups but comes into play here as well. The approach taken in this work is to track progress of the loss function over epoch/federated rounds and stop training when the loss converges to a stable value (see Supplementary Note 2).

In general, not all participants may be available at any one time in the federated setting. We therefore explore model quality as a function of client participation rate. Across the datasets, we find that only a minority of clients need to participate in any one round of federated learning (Figure 1). These sub-populations are sampled at random with replacement for each round. We see that just a 2% randomized participation rate achieves almost the same model quality as with full participation. This makes the federated setup quite robust to platform-independent bias caused by device dropout described in Supplementary Discussion 3.

Another dimension to consider is scalability of this approach in terms of total runtime of the federated experiment. Supplementary Figures 1, 15 and 17 show a relationship between the number of clients (again one client per example which is the most communication-intensive scenario), and the number of features (independent variables) captured in each training example. Across the domains, we see a linear relationship between the number of examples/clients and runtime. Furthermore, the dimensionality of the examples has no significant effect on runtime. This is because each client’s data is of relatively small size and therefore communication and computation overhead dominates the runtime. If high-bandwidth variables were used, such as video, the runtime would further increase by the transmission time on the network.

Since we have seen that example dimensionality has no significant effect on runtime within the datasets considered, we turn our attention to runtime until convergence as a function of the number of participants, using a synthetic dataset of size ranging from 1,000 to 10,000 clients (Supplementary Figure 1) and observe a strong linear relationship (R^2 of 0.997).

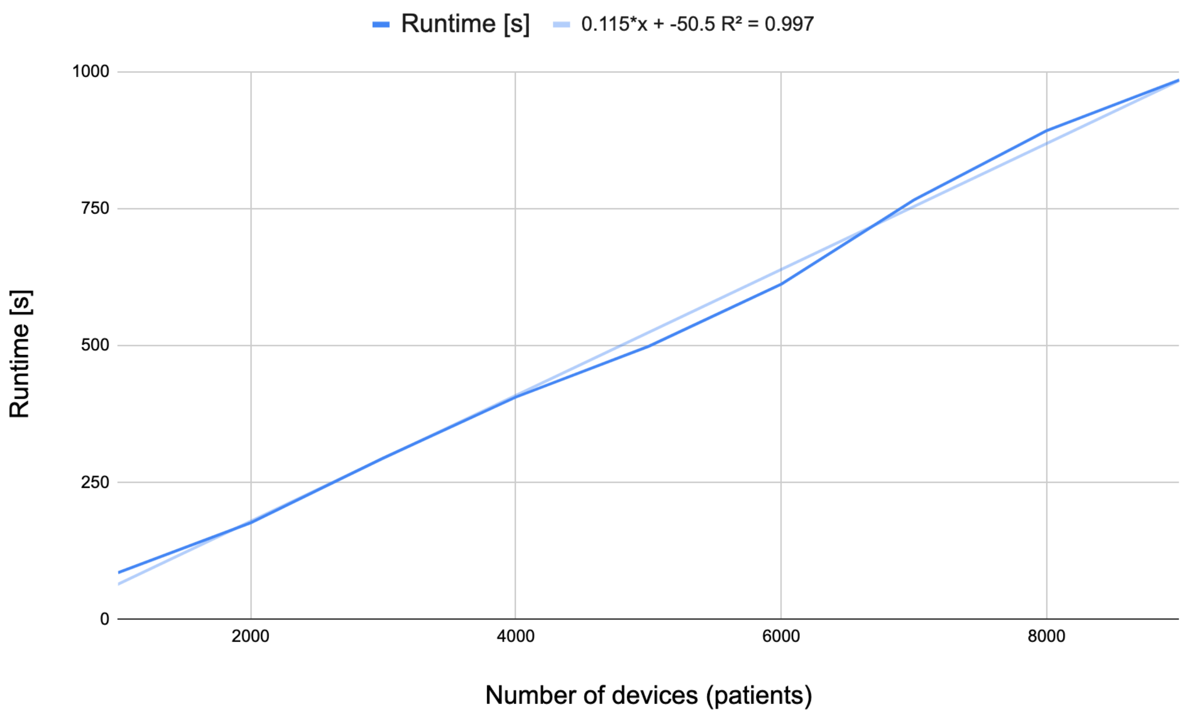

Supplementary Figure 1: Runtime until convergence as a function of the number of participants, ranging from 1,000 to a pool of 10,000. We observe a strong linear relationship (R^2 of 0.997).

Supplementary Discussion 1: Models

Logistic regression (LR)

Logistic regression is a generalized linear model with a logit link function given by

$$z = \frac{1}{1 + exp(-(\beta_0 + \sum_i \beta_i x_i))} \quad (1)$$

or equivalently

$$logit(z) = \beta_0 + \sum_i \beta_i x_i \quad (2)$$

where z is the dependent variable, **β**s are model coefficients to be learned, and in vector x are the predictor (independent) variables.

Commonly used implementations of LR are GLM in R, Statsmodels.api and sklearn in python, and SAS's PROC LOGISTIC. For parameter optimization, they use various techniques often optimized for the specific case of LR, such as iteratively reweighted least squares (IWLS, also called Fisher scoring) and coordinate-descent linear solver. We use GLM implemented in statsmodels (version 0.12.1) library as a "classical" centralized baseline for comparison.

To capture uncertainty in the fitted weights, rather than just point estimates, we use Tensorflow Probability layers. This is important because model stability and interpretability are often more important in health research than raw prediction accuracy. For example, in a plain regression model, we may learn that an H5N1 infection is six times more likely to be fatal in patients in their twenties compared to those under 10 years old (controlling for all other variables).[6] However, if this statistic has a large variance across participants or time horizons, its utility and resulting decisions may differ significantly. In the H5N1 example, the 95% confidence interval is quite broad: 2.05–18.53. Besides prediction, many health studies are interested in quantifying the association between exposure and outcome variables, adjusting for potential confounders. Therefore we also evaluate the validity of federated learning methods in terms of producing risk estimates comparable to those observed in centralized analysis. For each domain/dataset, we report fitted models, discuss uncertainty inherent in them, and analyze convergence (Supplementary Discussion 2).

Deep neural network (DNN)

Deep neural network (DNN) is a type of machine learning model architecture that uses multiple layers to progressively extract higher-level features from the raw input.[7] Over the last decade, DNN has shown its superior performance in fields including computer vision, speech recognition, natural and language processing, among other areas. Health data such as medical images and complex medical records (e.g. those in the MIMIC dataset) can largely benefit from this deep architecture for latent feature extraction.[8] However, compared to logistic regression that has a convex loss function, DNNs are in general non-convex and optimization algorithms are therefore not guaranteed to find the global minimum. Similarly to the logistic regression

models described above, here we focus on the reproducibility of centralized DNN-based health research in a federated learning setup.

We note that many common models, including logistic regression described above can be implemented in TensorFlow (TF) as a one-layer neural network with sigmoid activation. As we will see below, TF can express a broad spectrum of models and can be deployed in a variety of settings including on-device. We leverage this to bridge the classical and federated worlds. Specifically, we keep the TF model definition constant and only vary the training algorithm. This setup will allow us to test other model architectures in the future using the same infrastructure -- starting with LR as a special case but enabling general model specification within the TF language.

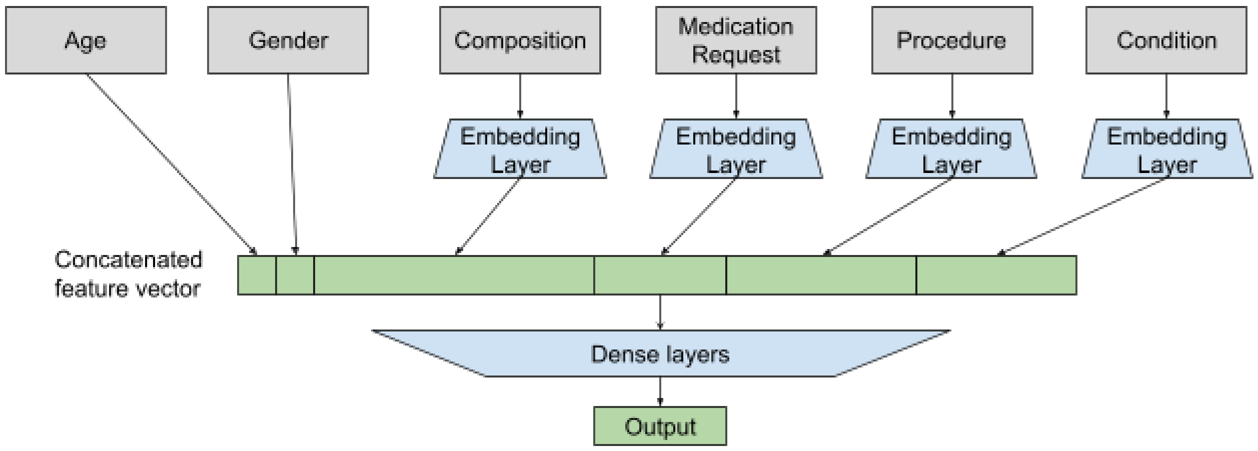

Supplementary Figure 2: Deep neural network model architecture for predicting inpatient mortality (Output) from sequence EHR data up to 24 hours after admission.

Supplementary Discussion 2: Further Results on Open Datasets

SARS-CoV-2 and Cancer

Based on the original work, the dataset contains three types of patients: a) Hospitalized, b) ICU admitted, and c) Deceased. For each type of patient, the analysis is divided into two parts based on: i) cancer interval and ii) cancer type. Cancer interval is the number of years a patient suffers from cancer before getting infected by Covid-19. There are different types of cancer reported in the dataset (see Supplementary Table 1).

<table>
<tr><td>Features</td><td>TF+DP</td><td>TFF</td><td>TFF+DP</td></tr>
<tr><td>Input features:
Age,Sex,Interval

Target: Hospital</td><td>Noise multiplier=69.3
Learning rate=0.7
Regularizer L2=1e-6
epsilon=0.25
delta=1e-5</td><td rowspan="2">Server optimizer
(Nadam, LR=0.15),
Regularizer L2=1e-6</td><td>Noise multiplier=1.3,
Regularizer L2=1e-6,
Adaptive clip LR=0.2,
Server optimizer
NADAM with LR=0.15</td></tr>
<tr><td>Input features::
Age,Sex,Interval

Target: ICU</td><td>Noise multiplier=50.3
Learning rate=20.0
Regularizer L2=1e-6
epsilon=0.335
delta=1e-5</td><td>Noise multiplier=1.3,
Regularizer l2=1e-7,
Adaptive clip LR=0.3,
Server optimizer
NADAM with LR=0.12</td></tr>
</table>

Input features:: Age,Sex,Interval<br><br>Target: Death	Noise multiplier=50.3<br>Learning rate=1.7<br>Regularizer L2=1e-6<br>epsilon=0.335<br>delta=1e-5	Server optimizer (Nadam, LR=0.2), Regularizer L2=1e-6	Noise multiplier=1.3, Regularizer l2=1e-7, Adaptive clip LR=0.3, Server optimizer NADAM with LR=0.4
Input features:: Age,Sex, Cancer type<br><br>Target: Hospital	Noise multiplier=50.3<br>Learning rate=1.5<br>Regularizer L2=1e-6<br>epsilon=0.335<br>delta=1e-5		Noise multiplier=1.3, Regularizer l2=1e-6, Adaptive clip LR=0.1, Server optimizer Nadam with LR=0.1
Input features:: Age,Sex, Cancer type<br><br>Target: ICU	Noise multiplier=50.3<br>Learning rate=32.0<br>Regularizer L2=1e-6<br>epsilon=0.335<br>delta=1e-5	Server optimizer (Nadam, LR=0.15), Regularizer L2=1e-6	Noise multiplier=1.3, Regularizer l2=1e-7, Adaptive clip LR=0.1, Server optimizer Nadam with LR=0.12
Input features:: Age,Sex, Cancer type<br><br>Target: Death	Noise multiplier=50.3<br>Learning rate=20.0<br>Regularizer L2=1e-6<br>epsilon=0.335<br>delta=1e-5		Noise multiplier=1.3, Regularizer l2=1e-7, Adaptive clip LR=0.1, Server optimizer Nadam with LR=0.3

Supplementary Table 1: Hyperparameter settings used for reproduction of the SARS-CoV-2 and Cancer experiments. In all of our experiments the hyperparameters of Logistic regression model are: a) Solver=lbfgs and b) C=1e7, and the hyperparameters of centralized tensorflow model are: a) Regularizer L2=1e-6 and Nadam optimizer with learning rate 0.15.

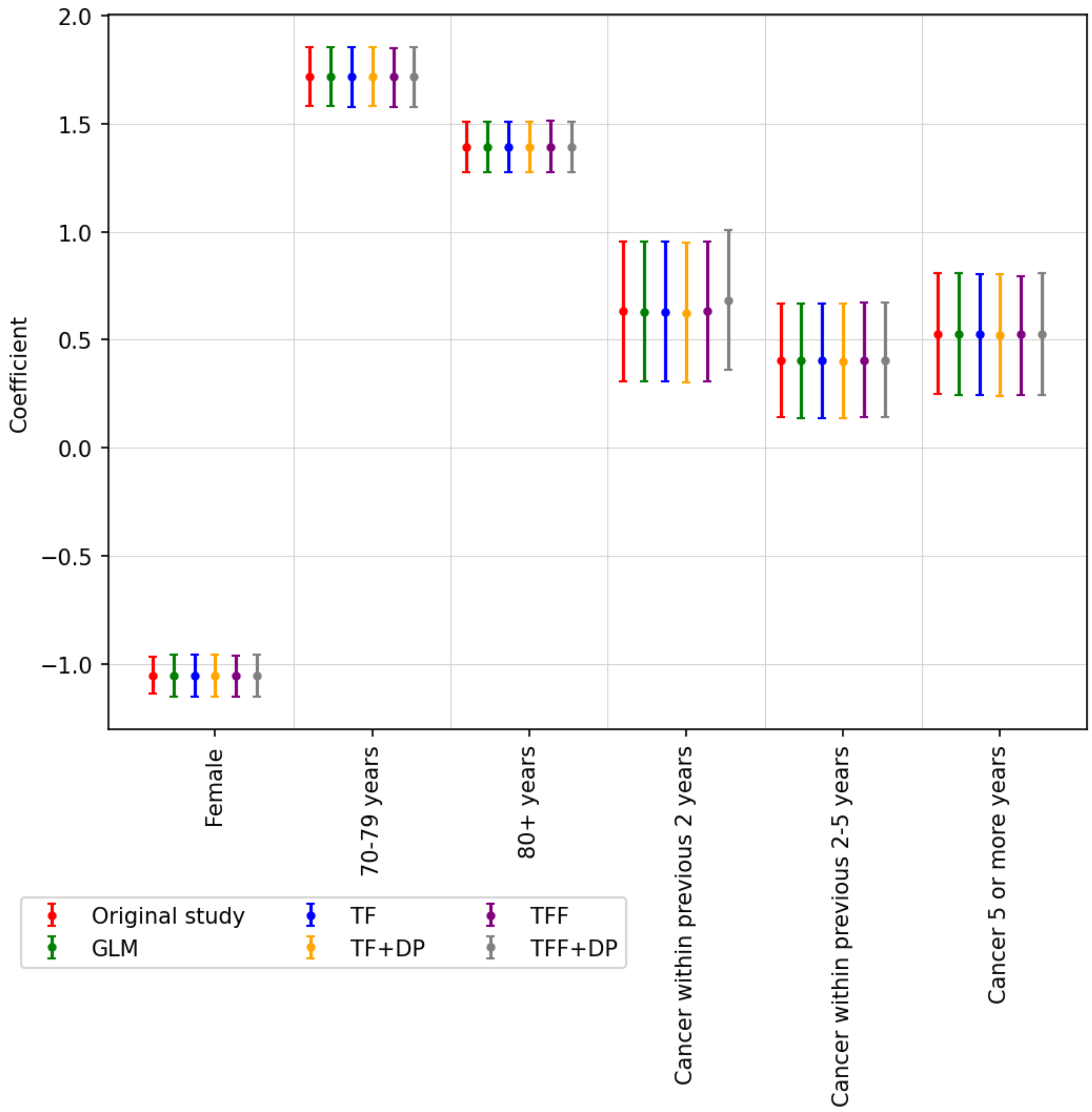

Supplementary Figure 3: Model coefficients and corresponding 95% confidence interval (CI) for hospitalized patients considering cancer interval. The coefficients reported in Rugge et al. (2020)[2]

are colored red and labeled ‘Original’. All of our models (centralized and federated) can estimate the coefficient that is very close to the ‘Original’ coefficient. The list of hyperparameters for this study is shown in Supplementary Table 1.

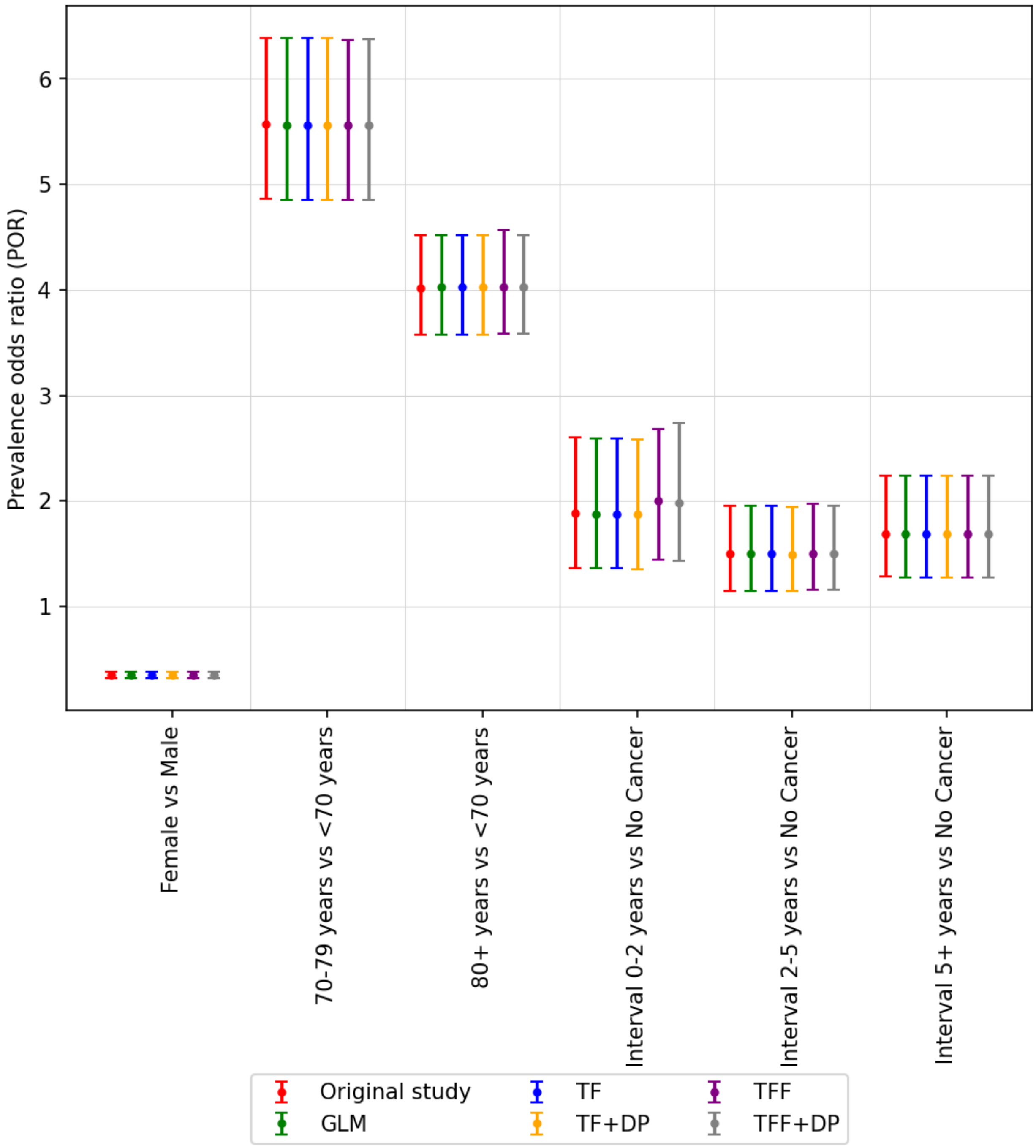

Supplementary Figure 4: The odds ratio and corresponding 95% confidence interval (CI) for hospitalized patients considering cancer interval. The odds ratio reported in Rugge et al. (2020)[2] is colored red and labeled 'Original'. All of our models (centralized and federated) can estimate the odds ratio that is very close to the 'Original' odds ratio. The list of hyperparameters for this study is shown in Supplementary Table 1.

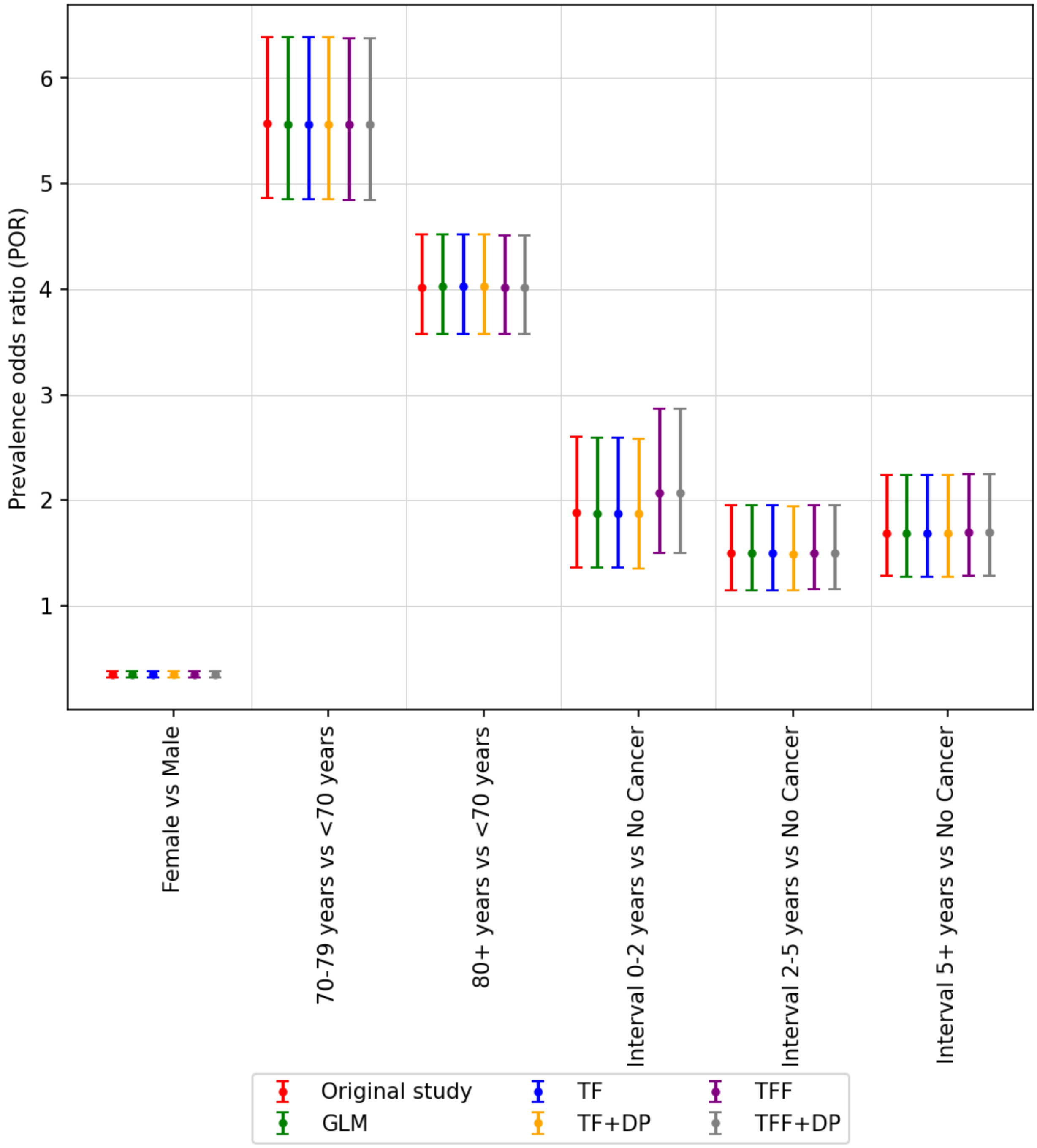

Supplementary Figure 5: The odds ratio is very close to the original study even when we created 100 random sized groups of patients (unit of federation) as shown here.

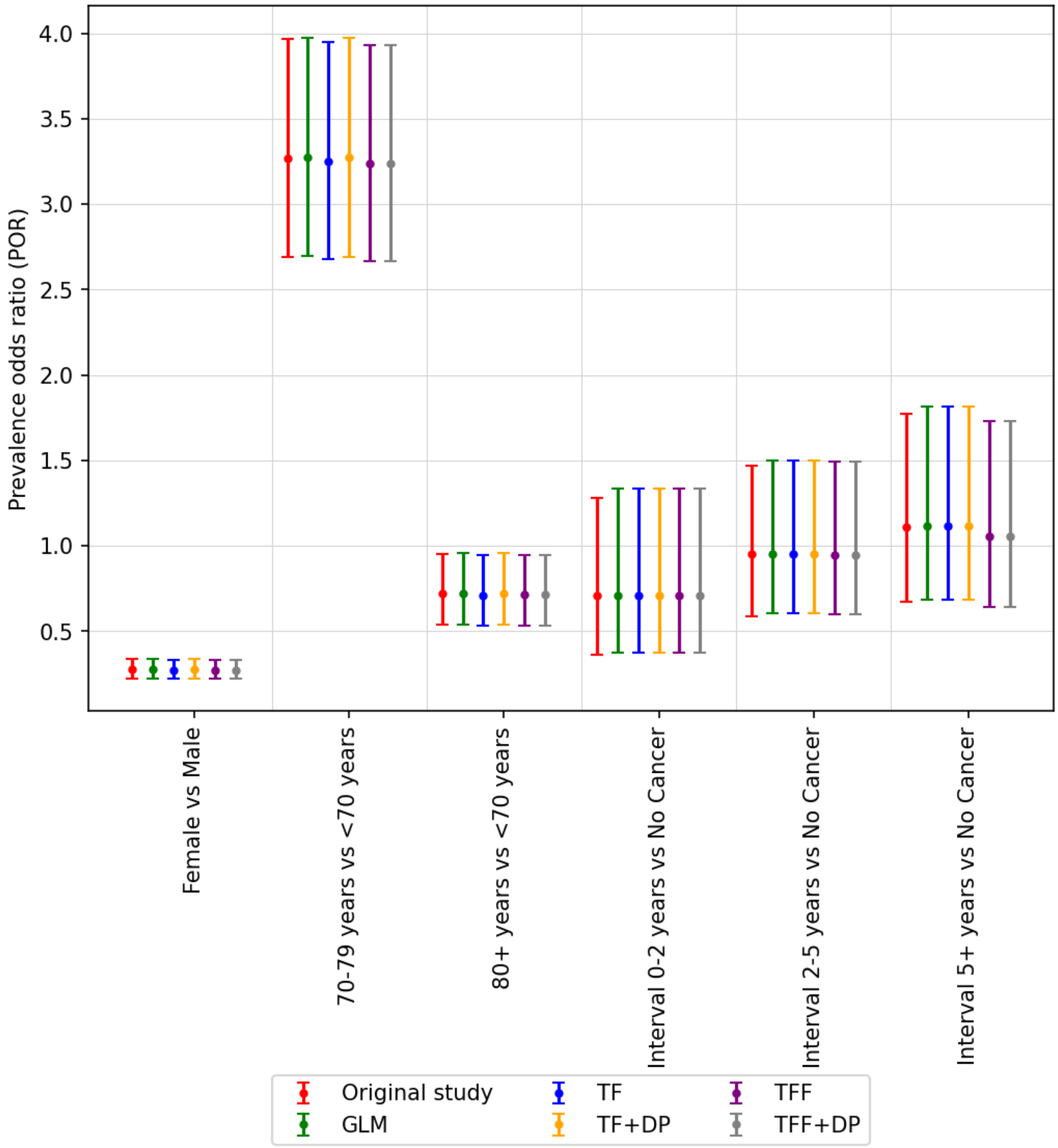

Supplementary Figure 6: The odds ratio and corresponding 95% confidence interval (CI) for ICU patients considering cancer interval. The odds ratio reported in Rugge et al. (2020)[2] is colored red and labeled 'Original'. All of our models (centralized and federated) can estimate the odds ratio that

is very close to the ‘Original’ odds ratio. The list of hyperparameters for this study is shown in Supplementary Table 1.

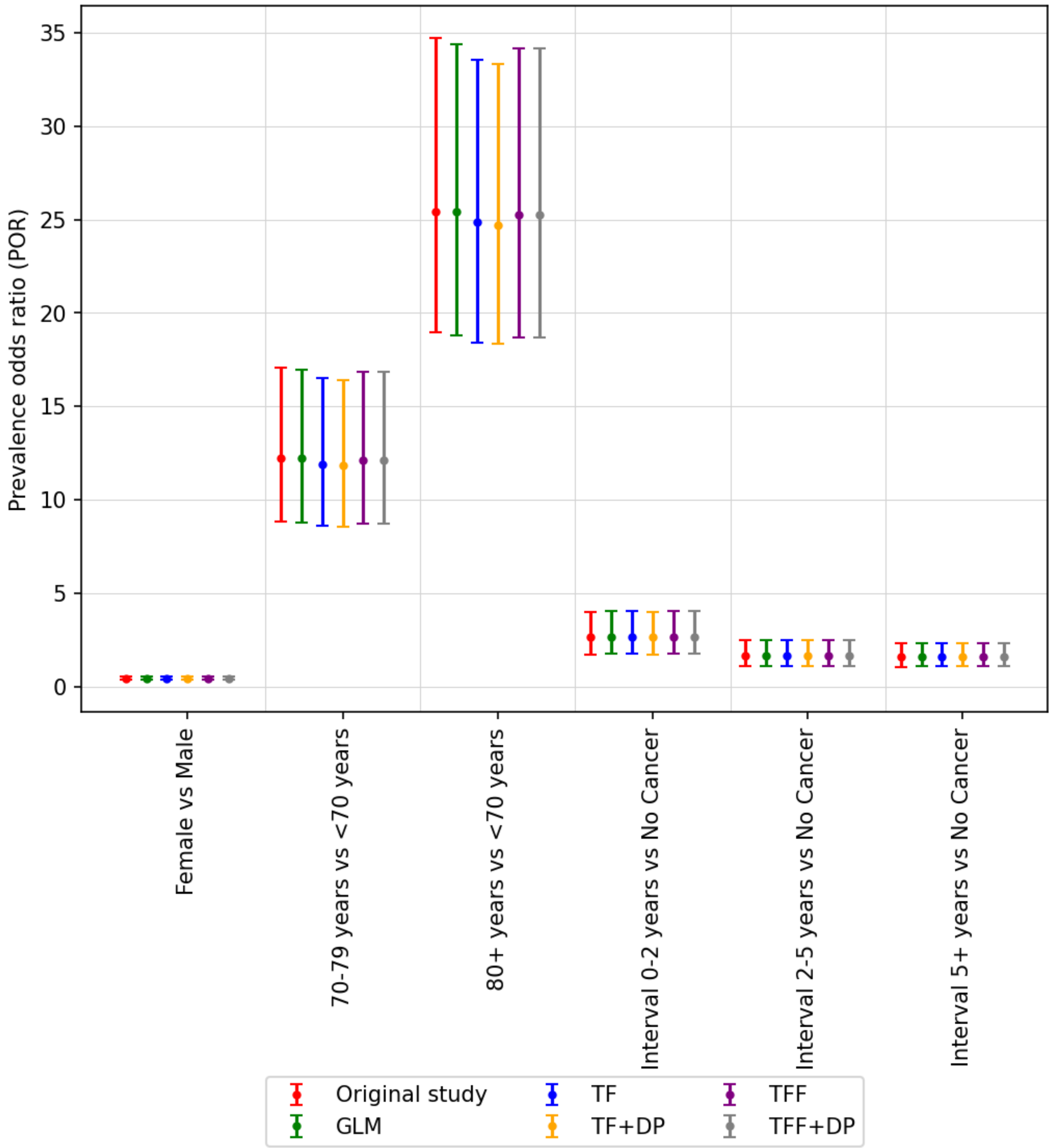

Supplementary Figure 7: The odds ratio and corresponding 95% confidence interval (CI) for dead patients considering cancer interval. The odds ratio reported in Rugge et al. (2020)[2] is colored red and labeled ‘Original’. All of our models (centralized and federated) can estimate the odds ratio that

is very close to the ‘Original’ odds ratio. The list of hyperparameters for this study is shown in Supplementary Table 1.

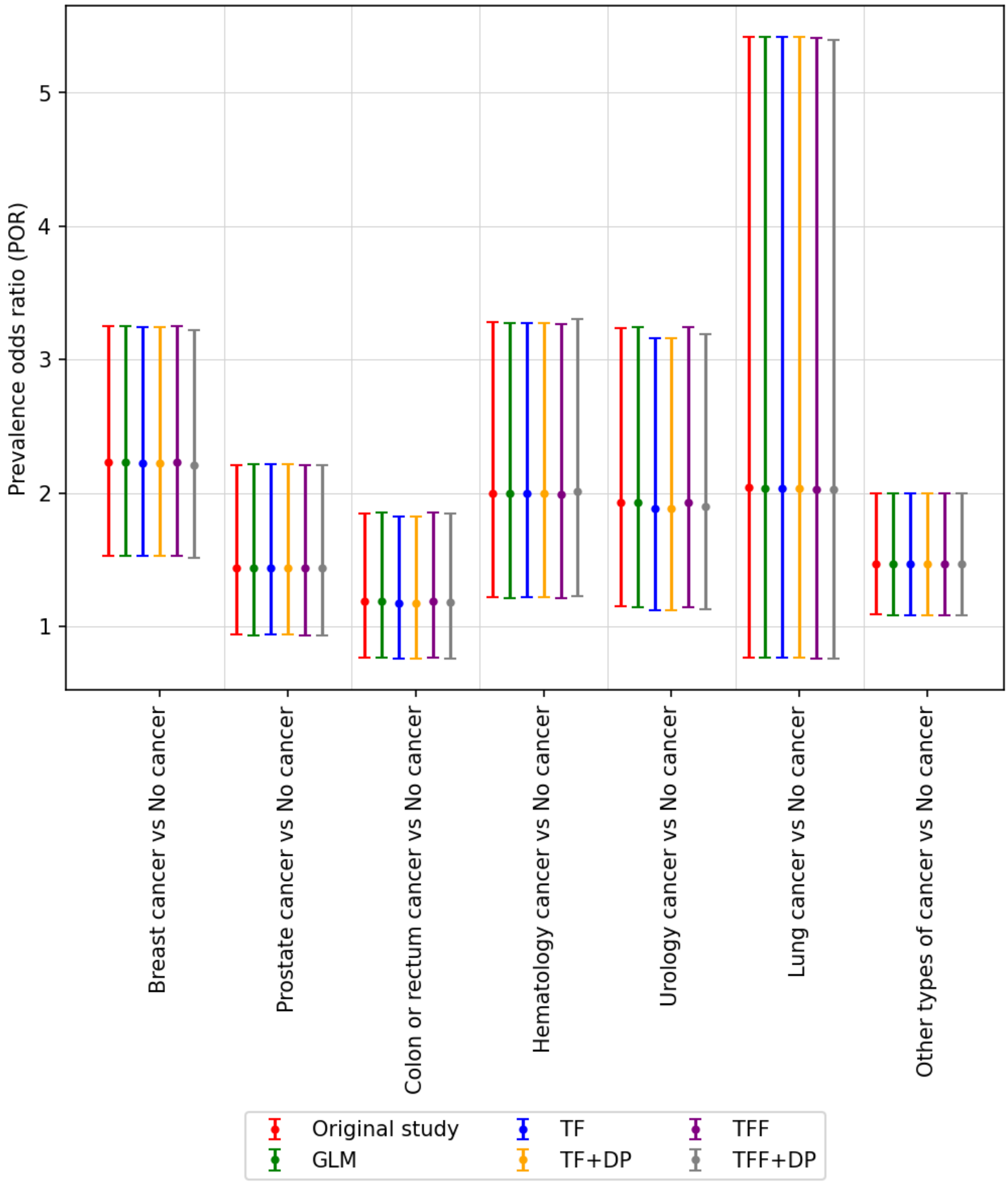

Supplementary Figure 8: The odds ratio and corresponding 95% confidence interval (CI) for hospitalized patients considering cancer type. The odds ratio reported in Rugge et al. (2020)[2] is colored red and labeled 'Original'. All of our models (centralized and federated) can estimate the

odds ratio that is very close to the ‘Original’ odds ratio. The list of hyperparameters for this study is shown in Supplementary Table 1.

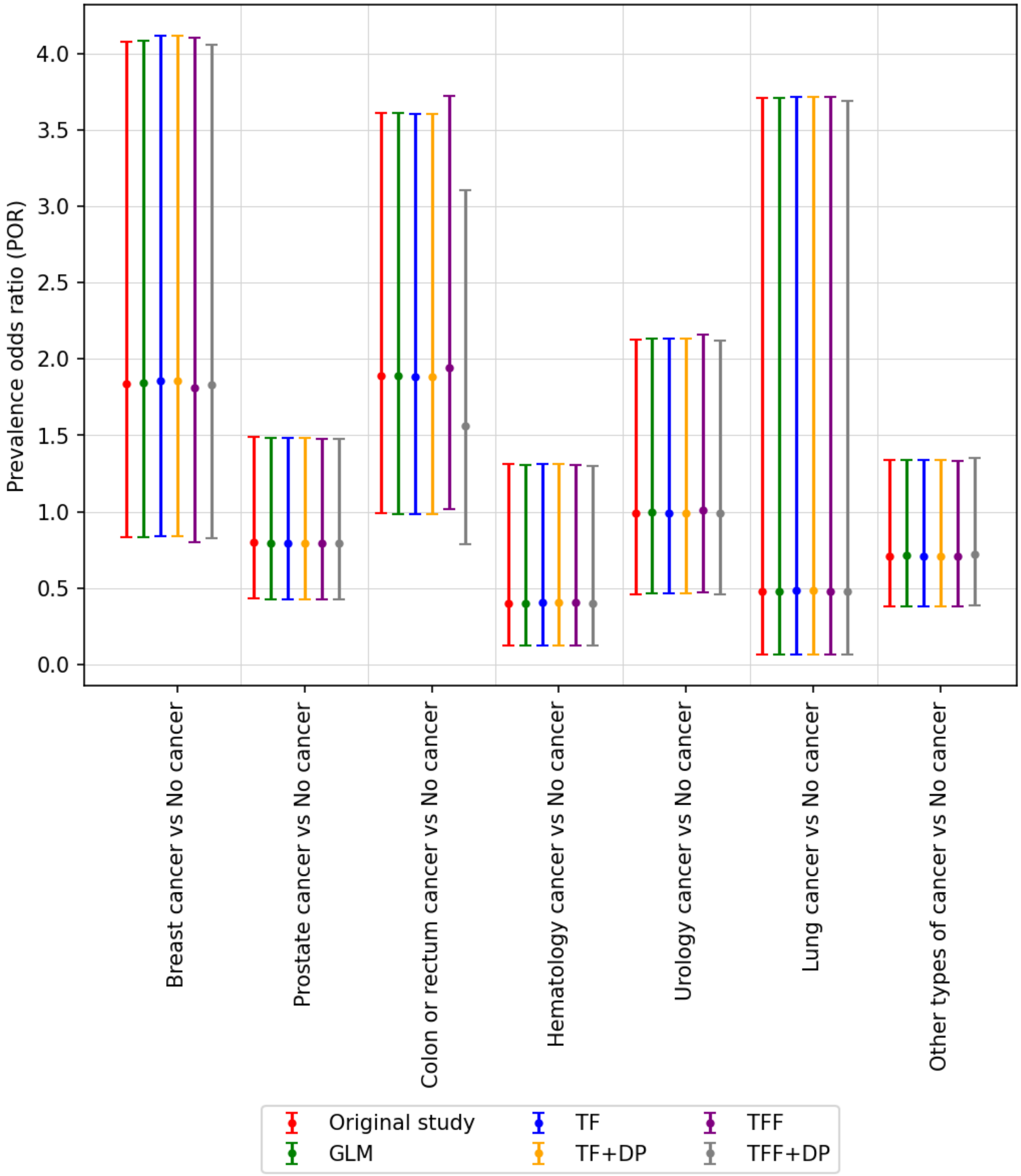

Supplementary Figure 9: The odds ratio and corresponding 95% confidence interval (CI) for ICU patients considering cancer type. The odds ratio reported in Rugge et al. (2020)[2] is colored red and labeled 'Original'. All of our models (centralized and federated) can estimate the odds ratio that is very close to the 'Original' odds ratio. The list of hyperparameters for this study is shown in Supplementary Table 1.

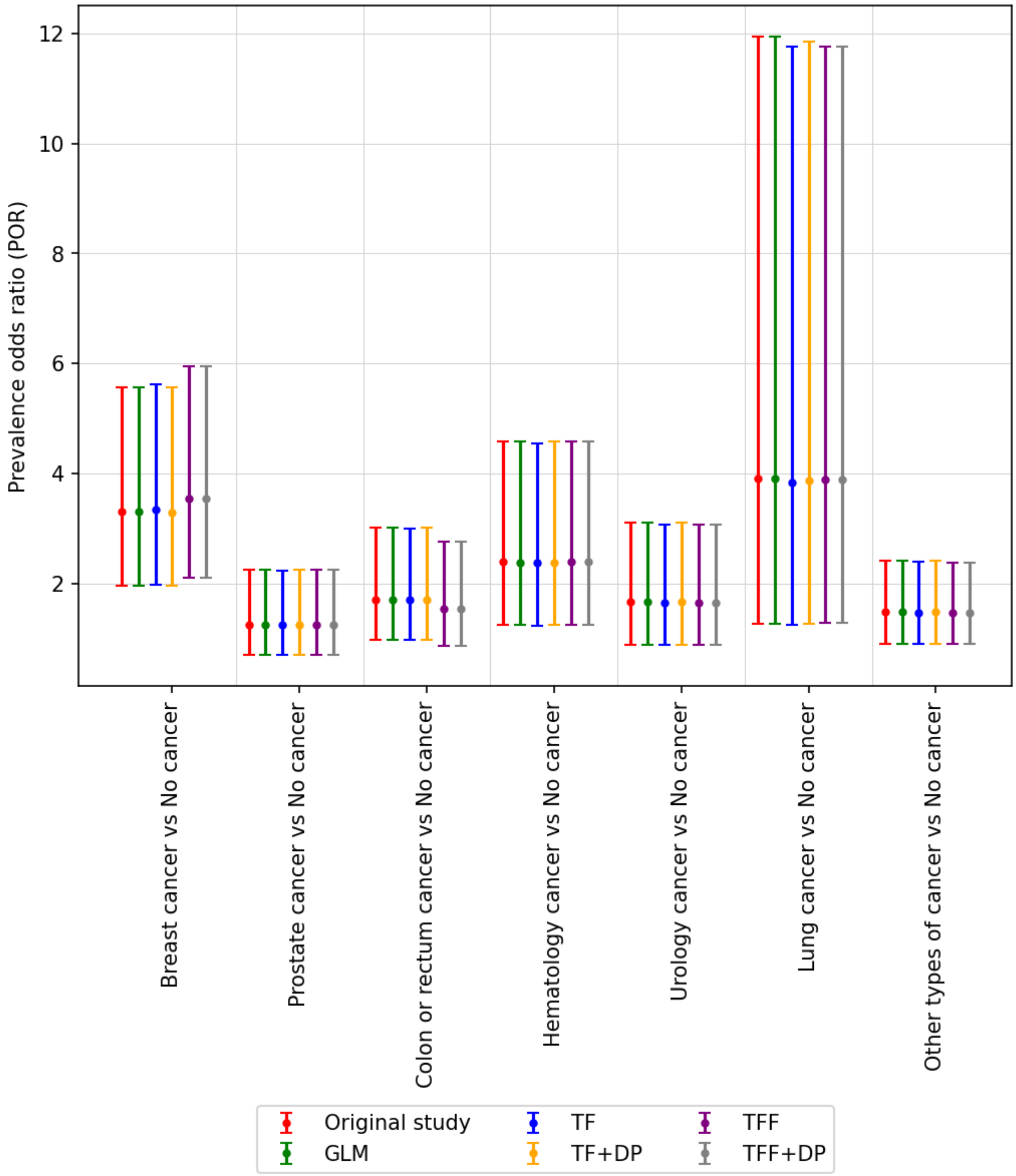

Supplementary Figure 10: The odds ratio and corresponding 95% confidence interval (CI) for dead patients considering cancer type. The odds ratio reported in Rugge et al. (2020)[2] is colored red and labeled 'Original'. All of our models (centralized and federated) can estimate the odds ratio that is

very close to the 'Original' odds ratio. The list of hyperparameters for this study is shown in Supplementary Table 1.

Avian influenza A (H5N1)

The Robert Koch Institute (RKI) avian influenza monitoring system is a publicly available epidemiological database established to track avian influenza infections in humans and animals around the world. This database includes 294 human cases from 12 different countries from 2006-2010, and it is used to predict risk of infection and mortality based on country, age, sex, time from symptom onset to hospitalization and exposure to poultry.[6]

Based on the prevalence odd ratio (pOR), Fiebig et al. (2011)[6] presented the following observations and the federated approach presented here reproduces all of them (Figure 3):

1. Odds of fatal outcome increased by 33% with each day that passed from symptom onset until hospitalization (OR: 1.33, 95% CI: 1.11–1.60).
2. The fatal outcome of both 10–19 year-olds and 20–29 year-olds is six times higher compared to 0-9 year-old children. The odds ratio of 10-19 years old vs 0-9 year-old children is 6.06 with CI: 1.89–19.48, whereas, the odds ratio of 20-29 years old vs 0-9 year-old children is 6.16 with CI: 2.05–18.53. On the other hand, the odds of fatal outcome is nearly five times higher in patients 30 years and older (OR: 4.71, 95% CI: 1.56–14.27) compared to 0-9 year-old children.
3. Using Indonesia as a reference, odds of dying were lower elsewhere, namely by 92% in Egypt (OR: 0.08, 95% CI: 0.03–0.22, p<0.001), by 81% in China (OR: 0.19, 95% CI: 0.04–0.90, p=0.036), and by 79% in Vietnam (OR: 0.21, 95% CI: 0.06–0.75, p=0.016), but not in the grouped remaining countries (OR: 0.23, 95% CI: 0.04–1.27, p=0.091).

Unlike for prediction tasks, in experiments on this data, we did not split the data for training and validation. All the observations were used to train the model as in the original study.[6]

To test various units of federation, we experiment with the extreme case of each patient being its own unit (Figure 3, Supplementary Figure 13), and with groups of patients (Supplementary Figure 14). Supplementary Figure 13 shows the ability of the federated approach to learn coefficients equivalent with the original work. Figure 3 shows an agreement in odds ratios across the models.

Feature	GLM	TF	TF+DP	TFF	TFF+DP
Hospitalization, Sex_female, Age_group, China, Egypt, Vietnam, and Other countries	Solver: lbfgs, C=1000	Regularizer L2=1e-7, Nadam with LR=0.26, epocs=300 with early stop	Noise multiplier=69.5 Learning rate=0.7 epocs=1300 epsilon=0.25 delta=1e-5	Server optimizer(nadam, LR=0.15), Regularizer L2=1e-6	Noise multiplier=1.3, Regularizer L2=1e-6 Adaptive clip LR=0.2, Server optimizer NADAM LR=0.25

Supplementary Table 2: Hyperparameter setting for H5N1 experiments.

Model convergence

Supplementary Figure 11 shows the training loss during our experiment considering all the observations to train the model. All the models converge to the minimum loss of 0.404. Tensorflow (TF) took 35 epochs and Tensorflow with differential privacy (TF+DP) took 400 epochs to converge. Tensorflow federated (TFF) took 230 epochs/rounds and Tensorflow federated with differential privacy (TFF+DP) took around 245 epochs/rounds to converge.

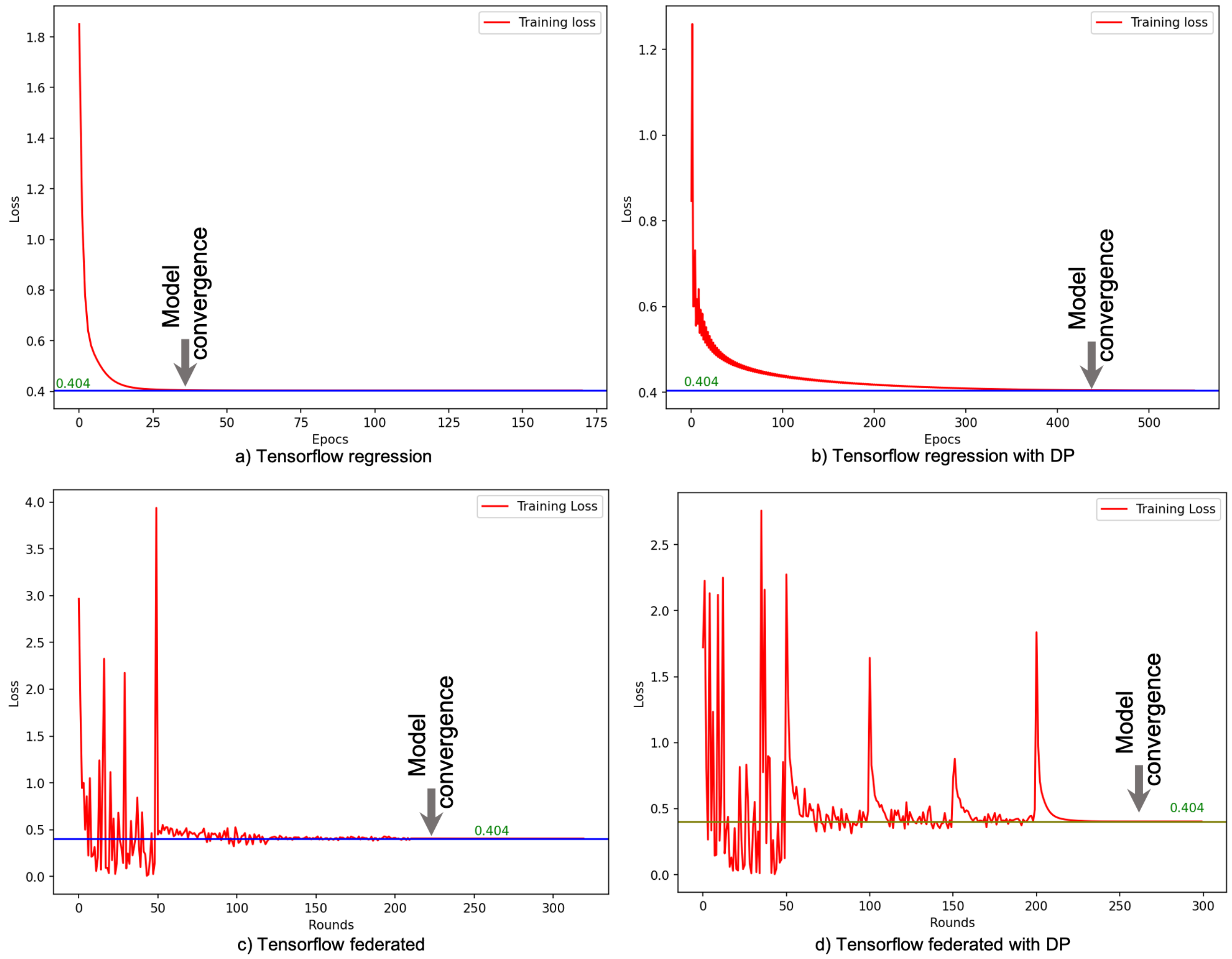

Supplementary Figure 11: Model convergence scenario.

Assign multiple data to a client

Besides assigning a single observation to a client, we did batch processing per client and each batch contains one country related observations. There are four countries in our experiments, therefore, we have four clients. The number of participants ranges from one to four which runs 100 rounds to each number of participants. According to the loss analysis (from Supplementary Figure 12), the minimum loss (0.404) of the model considering different optimizers is the same as unit federation.

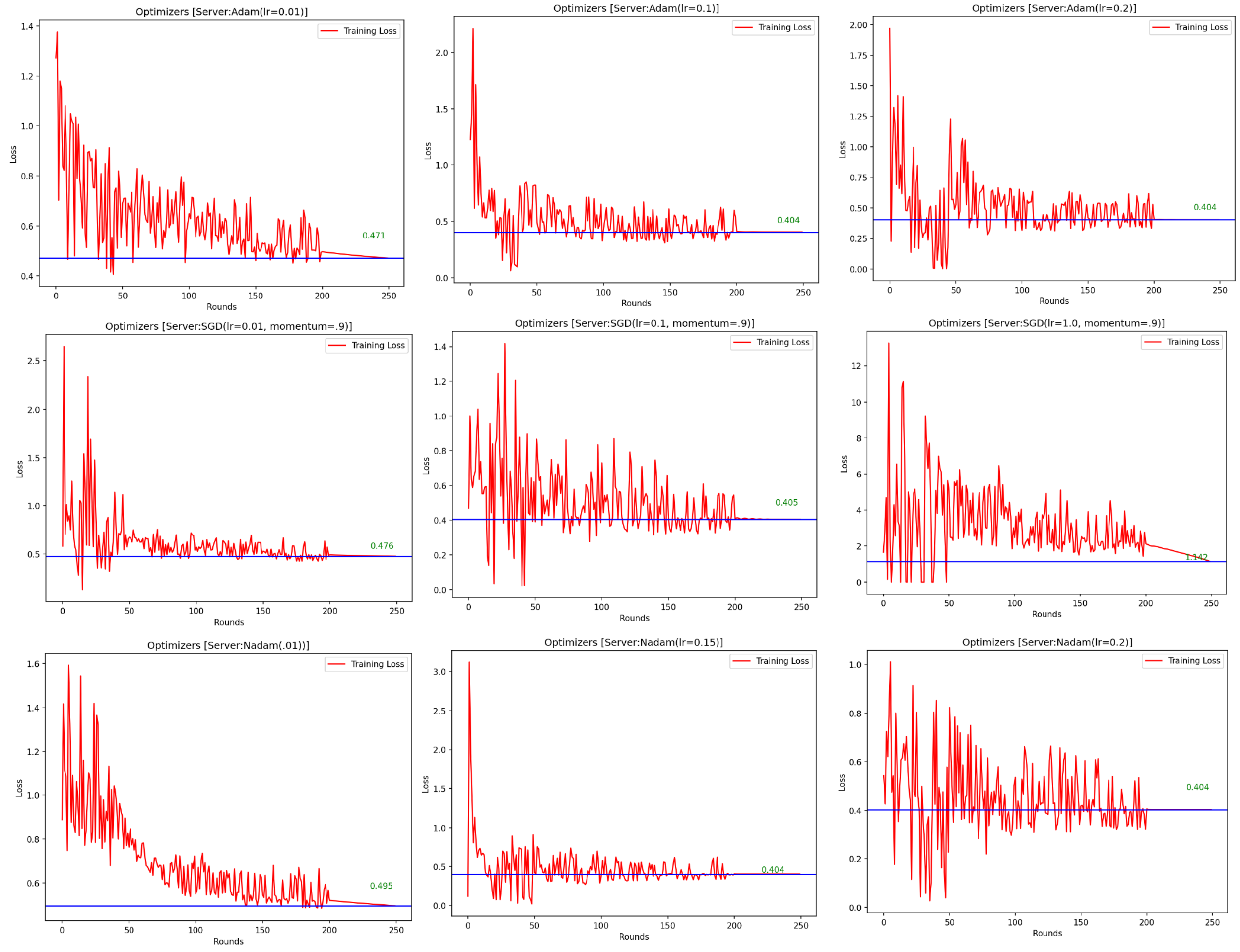

Supplementary Figure 12: Convergence for the TFF model is stable across several optimizer and hyperparameter settings.

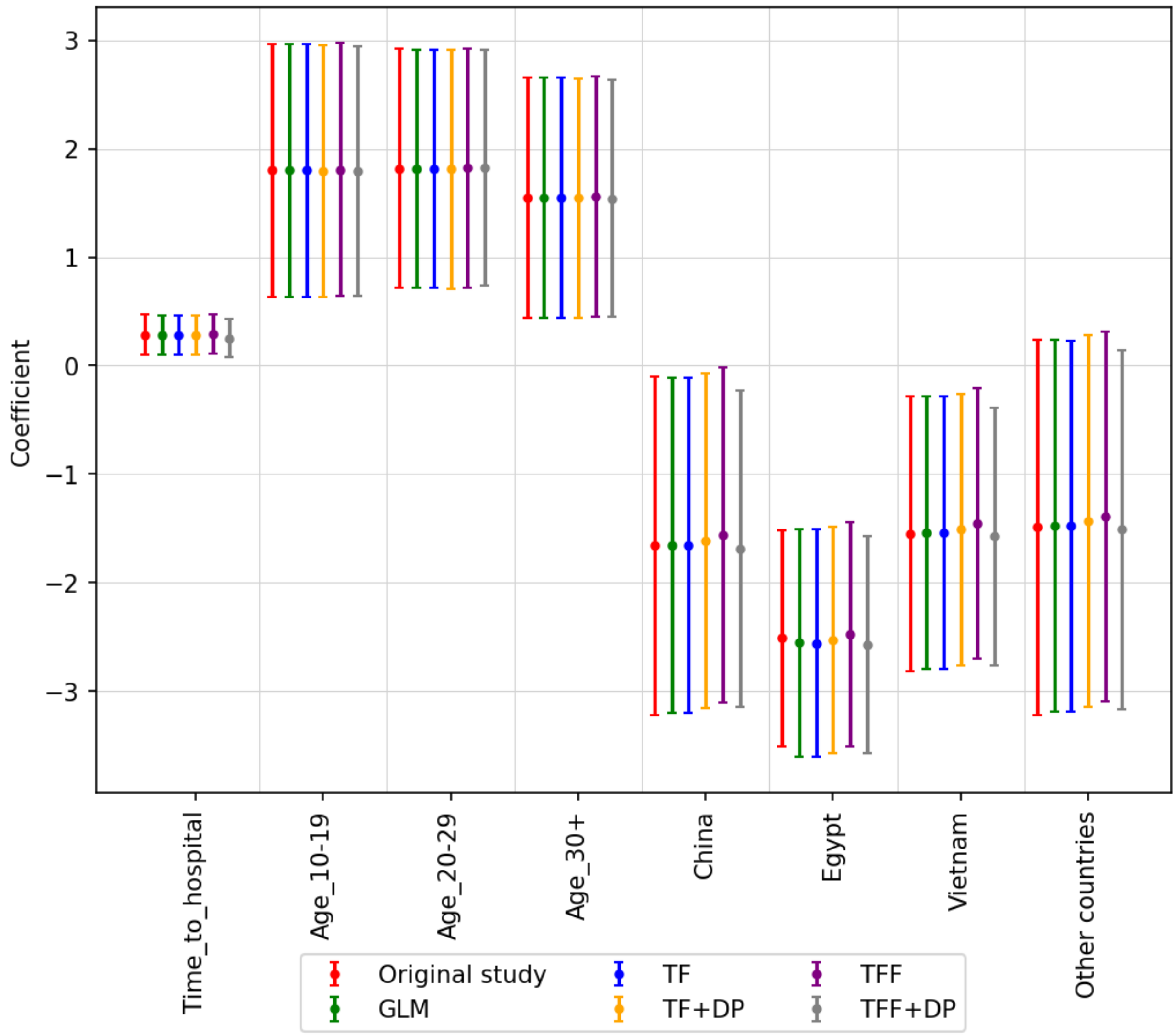

Supplementary Figure 13: The coefficient and corresponding 95% confidence interval (CI) for Avian influenza patients. The coefficients reported in Fiebig et al. 2011[6] are colored red and labeled 'Original'. All of our models (centralized and federated) can estimate the coefficients that are very close to the 'Original' odds ratio. The list of hyperparameters for this study is shown in Supplementary Table 2.

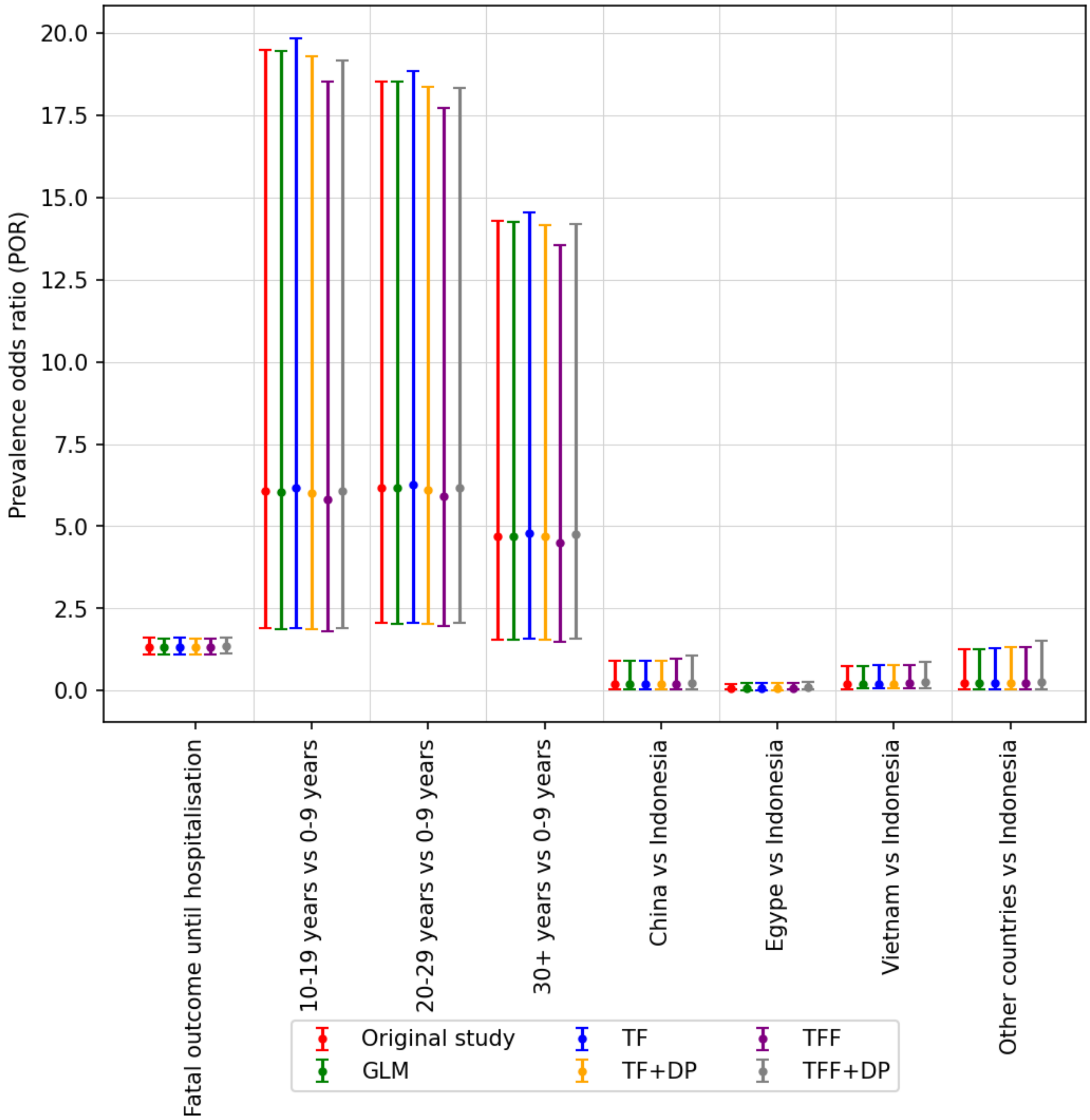

Supplementary Figure 14: The odds ratio is very close to the original study even when we grouped patients based on country (unit of federation) as shown here.

Diabetes

The Pima Indians Diabetes Dataset from the Kaggle machine learning data repository is a binary classification database involving females of Pima Indian heritage (https://www.kaggle.com/uciml/pima-indians-diabetes-database). This dataset is originally from the National Institute of Diabetes and Digestive and Kidney Diseases which began long-term longitudinal studies of the onset of diabetes in this population. The dataset is used to predict whether or not a patient will develop diabetes in 5 years time, based on eight attributes including age, body mass index, number of prior pregnancies, blood pressure, insulin and glucose levels.

Pioneering work on this task has achieved an AUC of 0.84 on predicting which individuals will develop diabetes 5 years in the future, with a neural network architecture with eight hidden nodes and one output node.[9] We replicate the same experimental setup and model architecture in TensorFlow Federated and augment it with central and local differential privacy. This reproduction yields a stronger AUC of 0.875 averaged over 10-fold cross-validation, while keeping each individual patient data local to their (emulated) storage device and providing strong (ϵ, δ)-differential privacy guarantees with central ϵ=0.736 and δ=10^{-5}, and local ϵ=11.8 and δ=10^{-9} per round. The higher AUC score of our implementation compared to the original study is likely due to advances in optimizing neural models that the field accomplished in the elapsed time and the use of regularization.

Like all other studies reproduced here, no differential privacy mechanism was used in the original work. By contrast, we include both central and local DP to evaluate model quality with this added layer of protection added. We observe that even with these fairly strong privacy guarantees added, model quality is minimally affected (mean AUC without DP is 0.881).

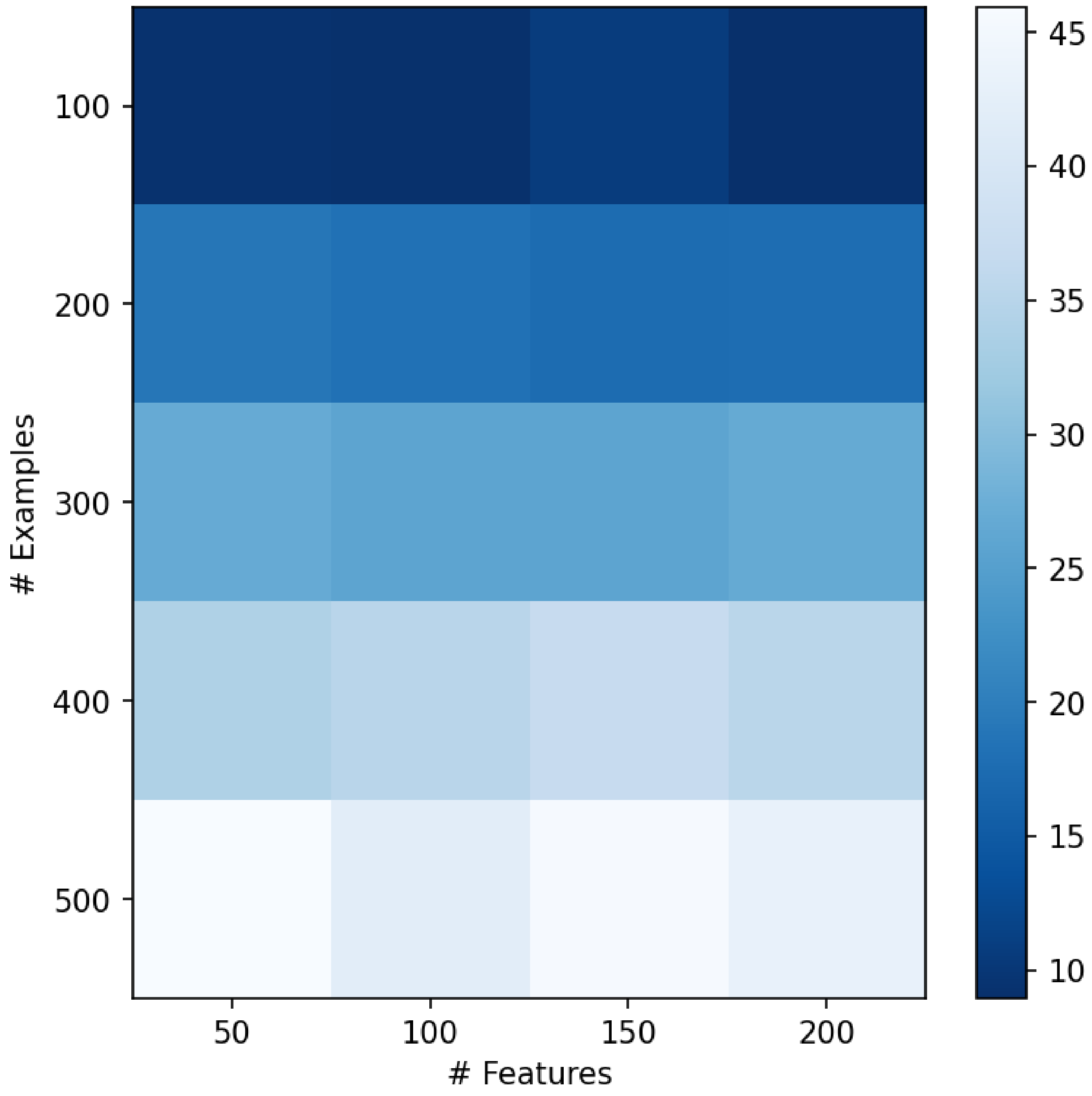

Supplementary Figure 15: Runtime of the federated learning process until convergence -- represented with shades of blue -- for the diabetes problem as a function of number of clients and number of features in each example. In this setting, the number of clients is equal to the number of examples since each participant contributes exactly one example. We see a linear relationship between the number of examples/clients and runtime. The dimensionality of the examples has no significant effect on runtime.

Bacteraemia

This bacteremia database involves 159 case-controlled cases of bacteraemia occurring among those of age 17 or over at four hospitals in Queensland and New South Wales, Australia between 1998 and 201.[10] The data is used to predict risk factors associated with relapsed infection in patients with *Enterobacer* bacteraemia, based on multiple factors including age, sex, location, source of infection, hospital location, co-morbid conditions, and many other clinical factors.

In the original study, the authors use multiple univariate logistic regression models to analyze the significance of the effects of clinical variables on relapsed Enterobacter bacteraemia. With a significance level of 0.05 among reported variables, Medical Service, Source (of infection) and Immune suppression are determined as significant variables. We replicate the models using GLM (Statsmodels.api), Tensorflow Probability (TF-Centralized) and Tensorflow Probability with Federated Learning (TF-Fed-Patient). Figure 4 shows the coefficients and their confidence intervals of all variables across tested models. The GLM models are consistent with the original study, where all of the above variables are significant and the rest are not. TF-Centralized and TF-Fed-Patient show narrower confidence intervals of their coefficients. The only difference is the significance of Acquisition status, where TF-Centralized and TF-Fed-Patient models determine it is significant and GLM does not. The p-value of the variable reported in the original study and our GLM model is 0.06, which is very close to the borderline of significance (0.05) and

hence, narrower CIs produced by TF-Centralized and TF-Fed-Patient consider it as borderline significant. With such a small difference (0.06 vs. 0.05), the conclusion of significance is expected to be sensitive to computational processes discussed in Supplementary Discussion 5 and highlights a sensitivity and interpretation challenge for the broader field of statistical modeling.

Azithromycin in Infants

The *Macrolides Oraux pour Réduire les Décès avec un Oeil sur la Résistance* (MORDOR) community-randomized study dataset is used to describe adverse events associated with azithromycin use in infants from 30 communities in Niger. The dataset includes 1,712 infants aged 1 to 5 months at time of treatment with azithromycin or placebo between January 2015 to February 2018. The dataset includes adverse events, age, sex, community and whether there were recent health issues prior to treatment.[11]

The original study evaluates the significance of side effects of azithromycin treatment on infants from 1 to 5 months old. The authors build generalized linear models on three different major target outcomes: if a child has any health problem, has to go to a clinic, or has any adverse event. The sole independent variable used in all models is the treatment/placebo indicator. The original study analyzes the significance of the GLMs to conclude that the correlations between the independent variable and the dependent variables are not statistically significant and hence, fail to reject the null hypothesis.

To replicate the results of the original study, we build logistic regression models using different methods: GLM (Statsmodels.api), Tensorflow Probability (TF-Centralized) and Tensorflow

Probability with Federated Learning (TF-Federated). We calculate the confidence intervals of the coefficients of the independent variable (treatment/placebo) in all models and examine the significance of the coefficients at 95% confidence (Supplementary Figure 16). Differential privacy does not significantly change the coefficients. In all models, the confidence intervals all contain 0 and hence all models fail to reject the null hypothesis. All conclusions are consistent with the original study.

We note that in Logistics Regression learning, there could be multiple equivalent optimums in the parameter space that could achieve the same value of the loss function. We notice that different learning methods may converge to different parameters that give rise to the same loss, which explains why their coefficients are not the same across methods. The important takeaway from this is even with different coefficient sets, the conclusion of the significance of these coefficients remains unchanged.

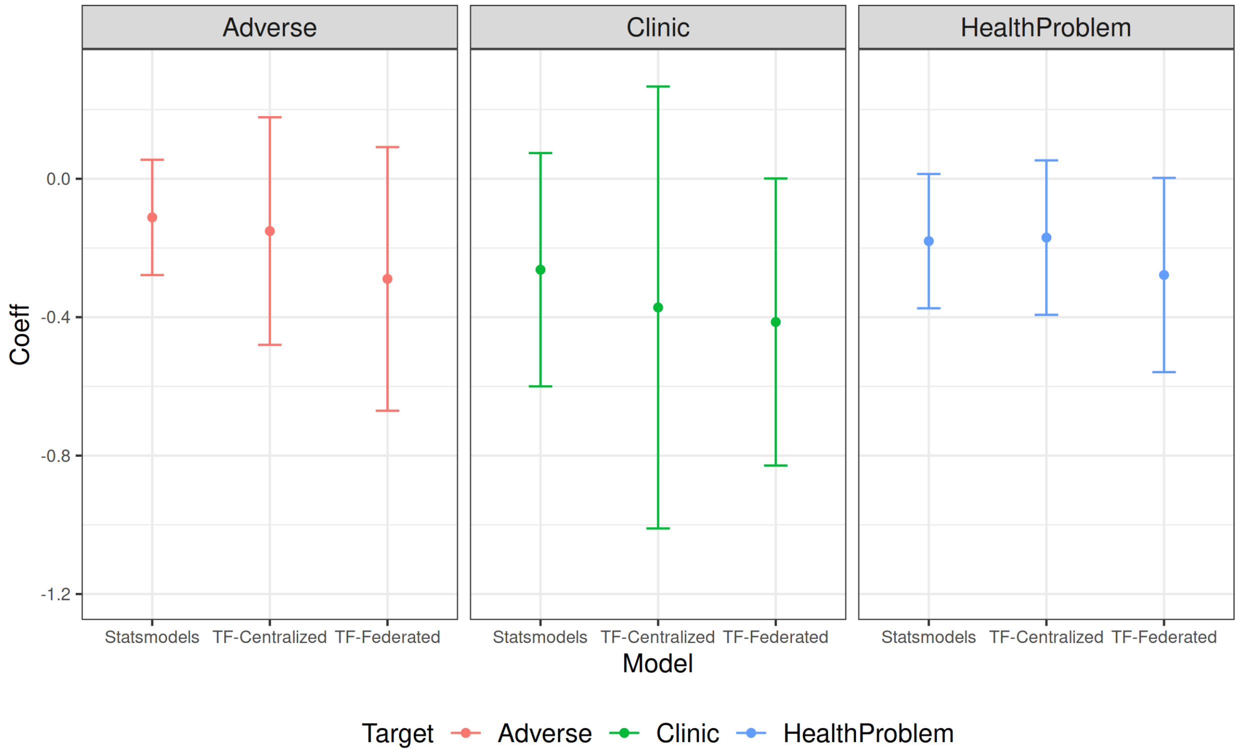

Supplementary Figure 16: Statsmodels.api (GLM), Tensorflow Probability (TF-Centralized) and Tensorflow Probability with Federated Learning (TF-Federated). We calculate the confidence intervals of the coefficients of the independent variable (treatment/placebo) in all models and examine the significance of the coefficients at 95% confidence. All models fail to reject the null hypothesis and therefore all conclusions are consistent with the original study.

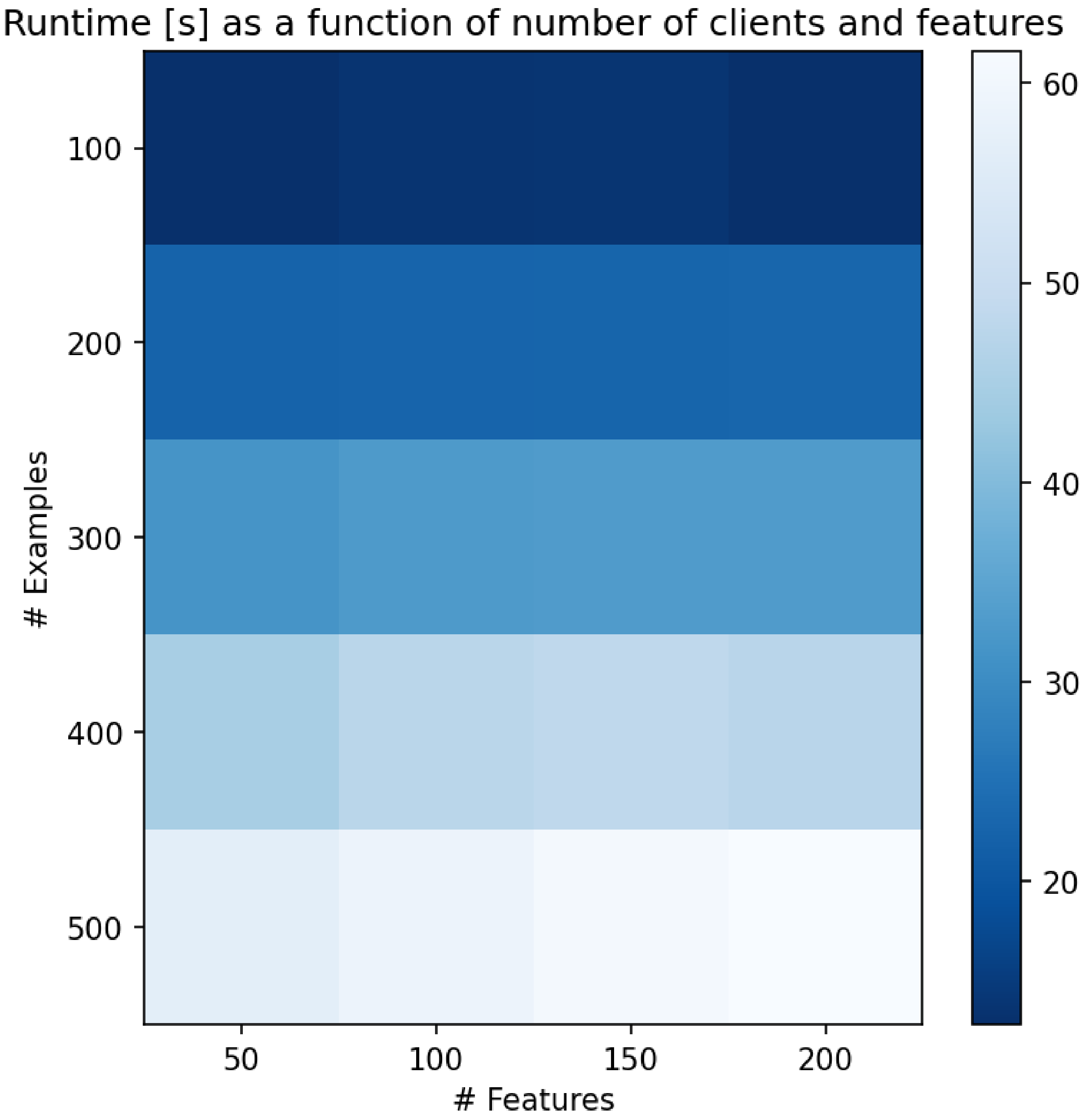

Supplementary Figure 17: Analogous to Supplementary Figure 15. Runtime of the federated learning process -- represented with shades of blue -- for the azithromycin in infants problem as a function of number of clients and number of features in each example. In this setting, the number of clients is equal to the number of examples since each participant contributes exactly

one example. We see a linear relationship between the number of examples/clients and runtime and no significant effect of example size.

Extrapulmonary tuberculosis

This Ghana Extra-pulmonary TB dataset is a medical records database of 3,704 TB patients diagnosed from June 2010 to December 2013 at 11 health facilities in Ghana.[12] The study participants include those 15 years and older with no prior history of TB. The study was conducted to understand the predictors of extrapulmonary TB compared to pulmonary TB such as HIV status and gender. The study also describes factors associated with mortality among patients with extrapulmonary TB (EPTB). The study dataset includes type of infection, health outcomes, age, sex, HIV status, site of infection, type of healthcare facility and year of diagnosis.

This is a multi-hospital dataset, where groups of data rows come from four different types of hospitals. Exploring multiple levels of federation reveals a challenge with grouping data at a hospital level, which has been a common de-siloing setup in the literature. Since one type of hospital (Teaching Hospital) in this dataset only contains examples of a specific type (HIV-positive), it creates a class-imbalance problem and the model converges to a different coefficient for HIV_Status variable (Supplementary Figure 18). Specifically, Teaching Hospital has 29% of all HIV-positive patients but only accounts for 12% of patients overall. Treating them as one unit may lower down the effect of HIV-positive variable to the dependent variable (EPTB). The conclusion stemming from this model's interpretation is still in line with all other methods, but lies outside of the 95% confidence interval.

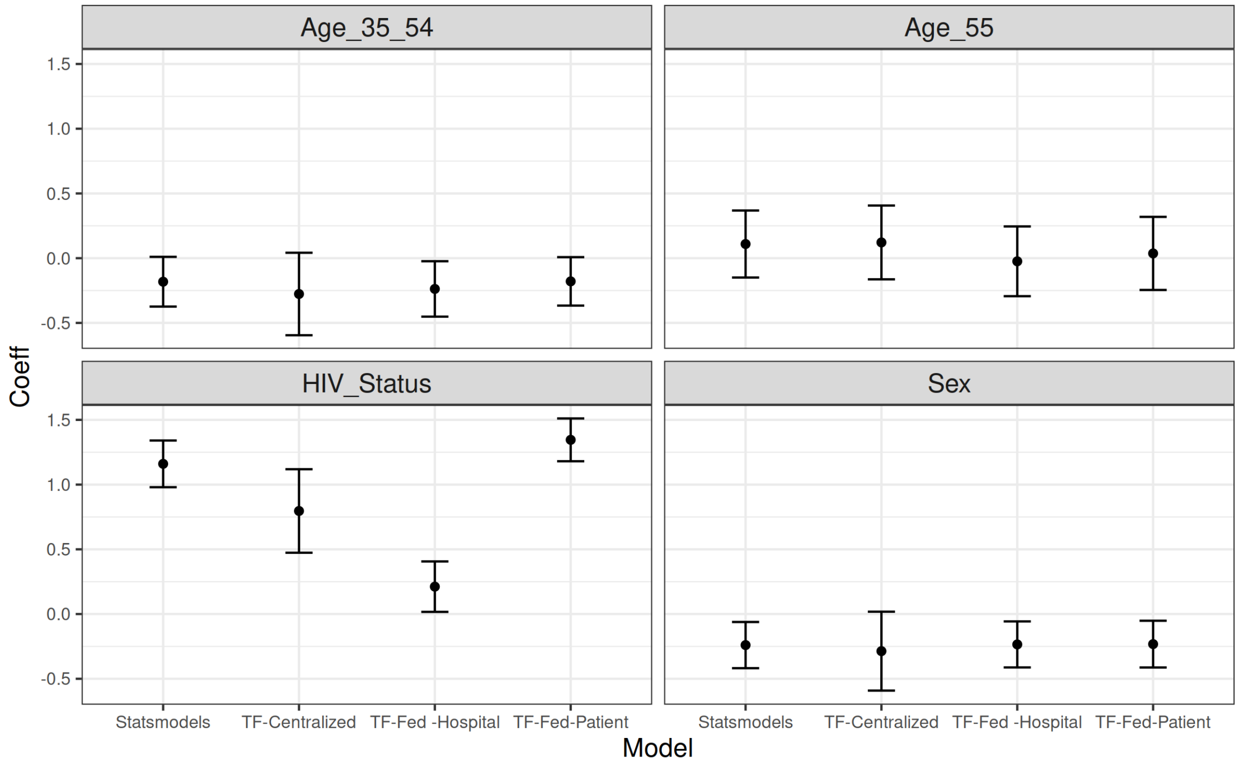

Supplementary Figure 18: The estimated coefficients and their 95% confidence intervals by different learning methods. Both Age variables are insignificant. Sex variables shows negative correlations, which means Female has larger risk to have EPTB than Male. HIV_status shows significant positive correlation, which indicates that patients with positive HIV have a significantly higher risk of having EPTB than negative HIV. All variable significance conclusions are consistent with the original study.

Supplementary Discussion 3: Limitations

Various sources of bias can enter a study and it is important to control for and mitigate it. Some types of bias -- platform independent bias -- are present irrespective of whether the study uses a

centralized data model or a federated one. For example, study drop out due to the subject deciding to leave the study or even dying, or selection bias as to who joins the study in the first place. Federated settings are subject to an additional type of bias -- platform-dependent bias. The subset of devices that contribute - and how often they contribute - is heavily influenced by fleet (i.e., population of participating devices) heterogeneity such as time and duration of availability; network bandwidth; processing time due to device capabilities and the amount of data to be processed; and survival bias due to network, low-memory, and charging state induced interruptions. Federated analytics can be used to monitor such events and patterns and react in the same way one would in a centralized study.

Since we reproduce prior studies in this work, we have to emulate a distributed dataset from the existing centralized tables produced by respective prior work. We do this by defining a federated averaging process that takes as input the original centralized dataset and segments it into units of federation, ranging from individual patients to progressively larger groups. In a live study, the platform-dependent bias outlined above would come into play. To explore it, we simulate various dropout scenarios in the results below and show the distributed computation is fairly robust. However, there are myriad dropout scenarios, and techniques to tackle them are only beginning to emerge. For example, federated analytics described below could be used to securely collect aggregate statistics over all study participants in order to detect various potential biases and be able to react to it and subsequently control for it. The techniques described here apply to the homogeneous federation units setting, where the output of federated computation from one silo is composable with the output from another silo. This is a necessary condition for FL to operate in the heterogeneous setting, but not a sufficient one.

Given the distributed nature of the federated approach, the hypotheses to be tested and the corresponding models need to be defined before the study starts and retrospective changes are by design not possible. However, pre-registering hypotheses ahead of time is a recommended practice that applies in any setting as it has been shown to help prevent data dredging and publication bias.[13] We note that a restart of a study with a new model is possible with the data recorded locally at participants' end points. Further, federated analytics can be used to perform initial aggregate data analysis, refine hypotheses or modelling approaches, and then run federated learning as described here.

While we evaluate only several classes of statistical models, they cover a large proportion of health research because they commonly arise in health studies. Additionally, federated learning has been shown to work on a broad range of models in general.[3] As we have seen however, the specific domain of clinical studies brings in unique challenges and future work will concentrate on testing additional model architectures.

Finally, this work focuses on horizontally-distributed data silos, where each participant has the same data schema and the rows of the full dataset are spread across the clients. While there are methods for record linkage,[1] we note that each study subject should always have access to *their own* data. Therefore, even if the data is split across multiple silos, the subject can download their data from all relevant silos they participate in, join them together vertically and then run the method described here.

Supplementary Discussion 4: Privacy Technologies

Secure Aggregation

Another accompanying technology is Secure Aggregation -- a secure multi-party computation (SMPC) protocol[4] that enables a centralized server to compute the sum of values submitted by several clients, without learning the values themselves. In the context of federated learning, each client's update can be represented as a tensor of values, and secure aggregation enables the federated learning orchestration server to compute the sum of many client's update, without accessing the values themselves (which are encrypted with keys that the server does not have).

Secure Aggregation provides two important privacy enhancements atop of baseline federated learning: it prevents the reversing of private data from individual clients' updates (since only a sum of many clients' updates is ever accessible, and not the updates themselves), and it can provide a measure of dissociation between clients and their updates (from the sum, it is impossible to determine which client contributed what components to it). Further, failure to execute the secure aggregation protocol results in the server learning no new information at all about clients, and the use of secure aggregation does not result in any quality/utility penalty to the learning process. Secure aggregation can also be used for variable standardization (e.g., z-score transformation). First, the required population-level statistics such as mean and standard deviation on a per-variable basis are computed. Then they can be distributed to participating devices which transform their local data before proceeding with the federated updates. In this work we leverage secure aggregation together with differential privacy to obtain

strong privacy guarantees at the level of local DP while recovering utility almost the same as with central DP.

Differential privacy (DP)

Differential privacy (DP) is a rigorous mathematical notion of information disclosure about individuals participating in computations over centralized or distributed dataset.[14] In order for a computation to be differentially private, no single entity can affect the results of the computation too much by joining or leaving the dataset. This definition implies that any one entity's contribution--no matter what it is--cannot be inferred from the differentially private result.

More formally, a computation is said to be differentially private if and only if, for any two datasets D_1 and D_2 that differ in only one element, the probability of any result S is almost the same. This difference can be at a participant-level (i.e., device user-level DP) where two adjacent datasets differ by all the training examples of a single study participant, or record-level DP where two adjacent datasets differ by 1 record (i.e. 1 training example). This work examines both levels of DP as we vary the units of federation (data silos) in a single unified framework. On one end of the spectrum we have exactly 1 training example per participant, in which case participant-level DP is equivalent to record-level DP. As we increase the silo size to groups of participants, we concentrate on participant-level DP as that is a stricter privacy guarantee notion.

One commonly used technical definition for a differentially private mechanism M is as follows:

$$Pr[M(D_1) = S] \leq e^{\epsilon} Pr[M(D_2) = S] + \delta$$

Under this formulation, known as (ϵ, δ)-differential privacy, ϵ characterizes the level of privacy for contributors, while δ can be thought of as bounding the probability of the privacy guarantee not holding. Smaller values of ϵ and δ imply better privacy guarantees.

Crucially, differential privacy *composes*: if two (ϵ, δ)-DP mechanisms are executed over the same data, the combined results are at worst (2ϵ, 2δ)-DP. This compositional property gives rise to the notion of a privacy "budget" which can be split across, for example, the iterative rounds of ML training algorithms like SGD, if each is individually differentially private.

Differential privacy can be used to mitigate the risk of model data memorization in machine learning.[15] By using training algorithms with known differential privacy properties, it is possible to compute the worst-case bound of having the private input data inferred from the model, regardless of the level of side-channel information available to the attacker.

Mechanisms for achieving differential privacy include adding uncertainty (strategically chosen noise) into the computation, bounding the contribution of any one entity and/or provably shuffling these entities' contributions. Most commonly, two models of differential privacy have been investigated: the central and local models. In central DP, the computation is done on full-fidelity data submitted by many entities, and then the differential privacy mechanism is applied. In contrast, in the local model each entity applies the differential privacy mechanism on its own data and the results are consequently aggregated. In a federated learning setting, these two models might be implemented by each client sending its update to the orchestrator as-is, and trusting it to apply the appropriate mechanism on the sum of many updated (the central

model), or by each client applying the DP mechanism to its update locally, before sending it along (the local model). Much health research to date does not incorporate DP mechanisms, but in this work we implement and run experiments with both central and local models of DP in combination with federated learning.

To obtain local differential privacy, we clip the gradients and then add Gaussian noise. We use the analytical calibration method for the Gaussian mechanism,[16] to calculate how much noise needs to be added locally to achieve a target local ϵ and δ. We choose a cryptographically small δ to ensure that the per-round local privacy loss random variable is almost always bounded. We leverage the fact that the addition of independent zero-mean Gaussian random variables is a Gaussian random variable and use moments accounting for the subsampled Gaussian mechanism[17] to derive the central ϵ for a target central $\delta = 10^{-5}$. We apply this methodology to all experiments involving DP in this work.

A recent advance in privacy research is a third “distributed” DP model that is well suited to federated learning applications. It combines features of the local model (each participating entity applies differential privacy locally) and the central model (the orchestrator post-processes encoded data to obtain accurate results.[18] However, instead of trusting the centralized orchestrator in adding noise, as the central model requires, the distributed DP model decentralizes the noise addition process and relaxes the trust requirements by using secure computations. The secure aggregation protocol described above is one example of this functionality. By combining secure aggregation with differential privacy, we can obtain a privacy guarantee almost the same as local DP while obtaining a utility almost the same as central DP.[1]

Supplementary Discussion 5: Reproducibility challenges

There is an increasing amount of evidence that many real-world problems in statistics and machine learning are under-specified.[19,20] This issue is ubiquitous and arises when there are different models -- each with a different set of parameters -- that explain validation data equally well. Since much of health research and epidemiology uses such modeling techniques, this challenge affects these fields as well. With under-specification, the models fail to capture generalizable inductive biases. As a result, traditional validation approaches cannot distinguish between them in terms of quality (e.g., ROC AUC, F1, accuracy) because they all perform equally well on the data available. However, when such models are deployed in practice on new unseen data, they often perform worse than would be expected based on their testing benchmark.[19]

More generally, the structural identifiability problem in dynamical systems concerns inferring unknown parameters of the model by given input-output data.[21] The perfect structural identifiability problem is one where the input-output relationships are noise free.[22] If the parameters of the dynamical system have infinitely many possibilities for the input-output data then the model is called identifiable. Practical identifiability deals with situations when the number of input-output data points are potentially noisy and few. The key points as regards to this paper are: (i) in general, one might not be able to uniquely reproduce identical parameters with limited training (input-output data), (ii) the issues when training a model using distributed methods is further complicated since various orders of training data might yield different outcomes.

In some of our experiments below, we encounter this problem as well. We find model fits that have equal loss value and equivalent validation ROC AUC, and yet differ in the values of model parameters (e.g., weights on independent variables). Prior work typically reported only one of such equivalent models, but in general there is a large number of them -- some differ only slightly in their parameter values, some are significantly different. This problem becomes even more acute when study conclusions are drawn from interpretation of model weights, as is common in much health research establishing a relationship between several independent variables and possible confounders and an outcome. The under-specified problem is a challenge for the broader field of statistical machine learning, an area of active research, and out of scope for this work to resolve. We mitigate the issue by reporting the multiple equivalent models we find in our reproduction experiment and compare them in a probabilistic framework with the original work that presented a single model in terms of distribution over parameter values, predictive power, and generalizability.

Supplementary Data: Data Repositories

SARS-CoV-2 and Cancer

Title: Malignancy in SARS-CoV2 infection

Version (Date): 4 (26.10.2020)

Author(s): Massimo Rugge, Manuel Zorzi, Stefano Guzzinati

Host: Figshare

Link: https://figshare.com/articles/dataset/Malignancy_in_SARS-CoV2_infection/12666698

DOI: https://doi.org/10.6084/m9.figshare.12666698.v4

License: CC0 1.0, https://creativecommons.org/publicdomain/zero/1.0/

Final Date Accessed: November 23, 2020

Modifications: The original data was not modified. Federated replica(s) of this data were produced as described in the manuscript.

Warranties/Endorsements: The original authors made no warranties regarding the use of this data nor endorsed the present manuscript or its findings.

Avian influenza A (H5N1)

Title: Avian influenza A(H5N1) in humans - line list

Version (Date): (12.08.2011)

Author(s): Lena Fiebig, Jana Soyka, Silke Buda, Udo Buchholz, Manuel Dehnert, Walter Haas

Host: Robert Koch Institut edoc server

Link: https://edoc.rki.de/handle/176904/7480

DOI: http://dx.doi.org/10.25646/7661

License: CC BY 3.0 DE, https://creativecommons.org/licenses/by/3.0/de/

Final Date Accessed: November 23, 2020

Modifications: The original data was not modified. Federated replica(s) of this data were produced as described in the manuscript.

Warranties/Endorsements: The original authors made no warranties regarding the use of this data nor endorsed the present manuscript or its findings.

Diabetes

Title: Pima Indians Diabetes Database

Version (Date): 1 (06.10.2016)

Author(s): UCI Machine Learning, a derivative of work produced by Smith, et al.

Host: Kaggle.com

Link: https://www.kaggle.com/uciml/pima-indians-diabetes-database

DOI: N/A

License: CC0 1.0, https://creativecommons.org/publicdomain/zero/1.0/

Final Date Accessed: November 23, 2020

Modifications: The original data was not modified. Federated replica(s) of this data were produced as described in the manuscript.

Warranties/Endorsements: The original authors made no warranties regarding the use of this data nor endorsed the present manuscript or its findings.

Electronic Medical Records

Title: MIMIC-III Clinical Database

Version (Date): 1.4 (04.09.2016)

Author(s): Alistair Johnson, Tom Pollard, Roger Mark

Host: PhysioNet

Host Citation: Goldberger, A., Amaral, L., Glass, L., Hausdorff, J., Ivanov, P. C., Mark, R., ... & Stanley, H. E. (2000). PhysioBank, PhysioToolkit, and PhysioNet: Components of a new research resource for complex physiologic signals. Circulation [Online]. 101 (23), pp. e215–e220.

Link: https://physionet.org/content/mimiciii/1.4/

DOI: https://doi.org/10.13026/C2XW26.

License: PhysioNet Credentialed Health Data License 1.5.0, https://physionet.org/content/mimiciii/view-license/1.4/

Final Date Accessed: November 23, 2020

Modifications: The original data was not modified. Federated replica(s) of this data were produced as described in the manuscript.

Warranties/Endorsements: The original authors made no warranties regarding the use of this data nor endorsed the present manuscript or its findings.

Heart Failure

Title: Heart failure clinical records Data Set

Version (Date): (05.02.2020)

Author(s): Davide Chicco, a derivative of work produced by Ahmad et. al

Host: UCI Machine Learning Repository

Link: https://archive.ics.uci.edu/ml/datasets/Heart+failure+clinical+records

DOI: N/A

License: CC BY 4.0, https://creativecommons.org/licenses/by/4.0/

Final Date Accessed: November 23, 2020

Modifications: The original data was not modified. Federated replica(s) of this data were produced as described in the manuscript.

Warranties/Endorsements: The original authors made no warranties regarding the use of this data nor endorsed the present manuscript or its findings.

Bacteraemia

Title: Risk factors for relapse or persistence of bacteraemia caused by Enterobacter spp.: a case-control study

Version (Date): 1.0 (18.01.2017)

Author(s): Patrick Harris

Host: Harvard Dataverse

Link: https://dataverse.harvard.edu/dataset.xhtml?persistentId=doi:10.7910/DVN/56NCVU

DOI: https://doi.org/10.7910/DVN/56NCVU

License: CC0 1.0, https://creativecommons.org/publicdomain/zero/1.0/

Final Date Accessed: November 23, 2020

Modifications: The original data was not modified. Federated replica(s) of this data were produced as described in the manuscript.

Warranties/Endorsements: The original authors made no warranties regarding the use of this data nor endorsed the present manuscript or its findings.

Azithromycin in Infants

Title: Replication Data for: MORDOR Infant Adverse Event Survey Data

Version (Date): 2.0 (01.11.2018)

Author(s): Ying Lin

Host: Harvard Dataverse

Link: https://dataverse.harvard.edu/dataset.xhtml?persistentId=doi:10.7910/DVN/MQYM5S

DOI: https://doi.org/10.7910/DVN/MQYM5S

License: CC0 1.0, https://creativecommons.org/publicdomain/zero/1.0/

Final Date Accessed: November 23, 2020

Modifications: The original data was not modified. Federated replica(s) of this data were produced as described in the manuscript.

Warranties/Endorsements: The original authors made no warranties regarding the use of this data nor endorsed the present manuscript or its findings.

Extrapulmonary Tuberculosis

Title: Replication Data for Extra-pulmonary tuberculosis: a retrospective study of patients in Accra, Ghana

Version (Date): 1.0 (02.08.2018)

Author(s): Sally-Ann Ohene

Host: Harvard Dataverse

Link: https://dataverse.harvard.edu/dataset.xhtml?persistentId=doi:10.7910/DVN/TA1OII

DOI: https://doi.org/10.7910/DVN/TA1OII

License: CC0 1.0, https://creativecommons.org/publicdomain/zero/1.0/

Final Date Accessed: November 23, 2020

Modifications: The original data was not modified. Federated replica(s) of this data were produced as described in the manuscript.

Warranties/Endorsements: The original authors made no warranties regarding the use of this data nor endorsed the present manuscript or its findings.

References

1. Zhu W, Kairouz P, Sun H, McMahan B, Li W. Federated heavy hitters with differential privacy. arXiv. 2019. https://github.com/tensorflow/ (accessed Nov 23, 2020).

2. Rugge M, Zorzi M, Guzzinati S. SARS-CoV-2 infection in the Italian Veneto region: adverse outcomes in patients with cancer. Nat Cancer 2020; 1: 784–8.

3. Bonawitz K, Eichner H, Grieskamp W, et al. TensorFlow Federated: Machine Learning on Decentralized Data. 2020. https://www.tensorflow.org/federated (accessed Nov 23, 2020).

4. Bonawitz K, Ivanov V, Kreuter B, Marcedone A, McMahan HB, et al. Practical secure aggregation for privacy preserving machine learning. In ACM Conference on Computer and Communications Security (ACM CCS), 2017.

5. Dozat T. Incorporating Nesterov Momentum into Adam. In ICLR Workshop, 2016. http://cs229.stanford.edu/proj2015/054_report.pdf.

6. Fiebig L, Soyka J, Buda S, Buchholz U, Dehnert M, Haas W. Avian influenza A(H5N1) in humans: New insights from a line list of World Health Organization confirmed cases, September 2006 to August 2010. Eurosurveillance 2011; 16: 19941.

7. LeCun Y, Bengio Y, Hinton G. Deep learning. Nature 2015; 521: 436–44.

8. Esteva A, Robicquet A, Ramsundar B, et al. A guide to deep learning in healthcare. Nat Med 2019; 25: 24–9.

9. Smith JW, Everhart JE, Dickson WC, Knowler WC, Johannes RS. Using the ADAP learning algorithm to forecast the onset of diabetes mellitus. In: Proceedings - Annual Symposium on Computer Applications in Medical Care. American Medical Informatics Association, 1988: 261–5.

10. Harris PNA, Peri AM, Pelecanos AM, Hughes CM, Paterson DL, Ferguson JK. Risk factors for relapse or persistence of bacteraemia caused by Enterobacter spp.: a case–control study. Antimicrob Resist Infect Control 2017; 6: 14.

11. Oldenburg CE, Arzika AM, Maliki R, Kane MS, Lebas E et al. Safety of azithromycin in infants under six months of age in Niger: A community randomized trial. PLoS Negl Trop Dis. 2018 Nov 12;12(11):e0006950.

12. Ohene S-A, Bakker MI, Ojo J, Toonstra A, Awudi D, Klatser P. Extra-pulmonary tuberculosis: A retrospective study of patients in Accra, Ghana. PLoS One 2019; 14: e0209650.

13. Hardwicke TE, Ioannidis JPA. Mapping the universe of registered reports. Nat Hum Behav. 2018 Nov;2(11):793-796. doi: 10.1038/s41562-018-0444-y.

14. Dwork C. Differential Privacy BT - Automata, Languages and Programming. In: Bugliesi M, Preneel B, Sassone V, Wegener I, eds. . Berlin, Heidelberg: Springer Berlin Heidelberg, 2006: 1–12.

15. Shokri R, Stronati M, Song C, Shmatikov V. Membership Inference Attacks Against Machine Learning Models. In: Proceedings - IEEE Symposium on Security and Privacy. 2017: 3–18.

16. Balle B, Wang YX. Improving the Gaussian mechanism for differential privacy: Analytical calibration and optimal denoising. In: 35th International Conference on Machine Learning, ICML 2018. 2018: 678–92.

17. Mironov I, Talwar K, Zhang L. Rényi Differential Privacy of the Sampled Gaussian Mechanism. arXiv. 2019. https://arxiv.org/abs/1908.10530 (accessed Dec 1, 2020).

18. Bittau A, Erlingsson Ú, Maniatis P, et al. PROCHLO: Strong Privacy for Analytics in the Crowd. In: SOSP 2017 - Proceedings of the 26th ACM Symposium on Operating Systems Principles. 2017: 441–59.

19. D'Amour A, Heller K, Moldovan D, et al. Underspecification Presents Challenges for Credibility in Modern Machine Learning. arXiv 2020; published online Nov 6. http://arxiv.org/abs/2011.03395 (accessed Nov 23, 2020).

20. Renard F, Guedria S, Palma N De, Vuillerme N. Variability and reproducibility in deep learning for medical image segmentation. Sci Rep 2020; 10: 13724.

21. Walter E, Pronzato L. On the identifiability and distinguishability of nonlinear parametric models. Math Comput Simul 1996; 42: 125–34.

22. Meshkat N, Sullivant S, Eisenberg M. Identifiability Results for Several Classes of Linear Compartment Models. Bull Math Biol 2015; 77: 1620–51.
