# Supplementary material for: Privacy-first health research with federated learning
Source: NPJ Digit Med. 2021 Sep 7;4:132. doi: 10.1038/s41746-021-00489-2 (PMC8423792; doi:10.1038/s41746-021-00489-2)
Supplement: Supplementary file 2 — Reporting Summary [file 41746_2021_489_MOESM2_ESM.pdf]

nature research

Corresponding author(s): Adam Sadilek (adsa@google.com) John Hernandez (johnbhernandez@google.com)

Last updated by author(s): Dec 7, 2020

Reporting Summary

Nature Research wishes to improve the reproducibility of the work that we publish. This form provides structure for consistency and transparency in reporting. For further information on Nature Research policies, see our Editorial Policies and the Editorial Policy Checklist.

Statistics

For all statistical analyses, confirm that the following items are present in the figure legend, table legend, main text, or Methods section.

n/a	Confirmed	
☐	☒	The exact sample size (*n*) for each experimental group/condition, given as a discrete number and unit of measurement
☒	☐	A statement on whether measurements were taken from distinct samples or whether the same sample was measured repeatedly
☐	☒	The statistical test(s) used AND whether they are one- or two-sided<br>*Only common tests should be described solely by name; describe more complex techniques in the Methods section.*
☐	☒	A description of all covariates tested
☒	☐	A description of any assumptions or corrections, such as tests of normality and adjustment for multiple comparisons
☐	☒	A full description of the statistical parameters including central tendency (e.g. means) or other basic estimates (e.g. regression coefficient) AND variation (e.g. standard deviation) or associated estimates of uncertainty (e.g. confidence intervals)
☐	☒	For null hypothesis testing, the test statistic (e.g. *F*, *t*, *r*) with confidence intervals, effect sizes, degrees of freedom and *P* value noted<br>*Give P values as exact values whenever suitable.*
☒	☐	For Bayesian analysis, information on the choice of priors and Markov chain Monte Carlo settings
☒	☐	For hierarchical and complex designs, identification of the appropriate level for tests and full reporting of outcomes
☒	☐	Estimates of effect sizes (e.g. Cohen's *d*, Pearson's *r*), indicating how they were calculated

Our web collection on statistics for biologists contains articles on many of the points above.

Software and code

Policy information about availability of computer code

Data collection

All data sources used in this manuscript are publicly available. Links for each dataset are available in Appendix A8. Code for the analysis is available at: [repository published long with the manuscript].

Data analysis

All data is available openly:
SARS-CoV-2 and Cancer
Rugge M, Zorzi M, Guzzinati S. Malignancy in SARS-CoV2 infection. 2020; published online Oct 26. DOI:10.6084/m9.figshare.12666698.v4. Accessed Nov 23, 2020.

Avian influenza A (H5N1)
Fiebig L, Soyka J, Buda S, Buchholz U, Dehnert M, Haas W. Avian influenza A(H5N1) in humans - line list. Publ. Serv. Robert Koch Inst. 2011; published online Aug 12. DOI:10.25646/7661. Accessed Nov 23, 2020.

Diabetes
UCI Machine Learning. Pima Indians Diabetes Database. Kaggle.com. 2016. https://www.kaggle.com/uciml/pima-indians-diabetes-database (accessed Nov 23, 2020).

Electronic Medical Records
Johnson A, Pollard T, Mark R. MIMIC-III Clinical Database v1.4. PhysioNet. 2016. doi.org/10.13026/C2XW26. Accessed Nov 23, 2020.

Heart Failure
Chicco D. Heart failure clinical records Data Set. UCI Machine Learning Repository. 2020. https://archive.ics.uci.edu/ml/datasets/Heart+failure +clinical+records (accessed Dec 1, 2020)

Bacteraemia

Harris P. Risk factors for relapse or persistence of bacteraemia caused by Enterobacter spp.: a case-control study, https://doi.org/10.7910/DVN/56NCVU, Harvard Dataverse, V1 (2017). Accessed Nov 23, 2020.

Azithromycin in Infants
Lin Y. Replication Data for: MORDOR Infant Adverse Event Survey Data. Harvard Dataverse, V2; doi:10.7910/DVN/MQYM5S. Accessed Nov 23, 2020.

Extrapulmonary Tuberculosis
Ohene S-A. Ghana Extra-pulmonary TB data set.tab. Replication Data Extra-pulmonary Tuberc. a Retrosp. study patients Accra, Ghana. doi:10.7910/DVN/TA1OII/ZSVFGO. (2019). Accessed Nov 23, 2020.

For manuscripts utilizing custom algorithms or software that are central to the research but not yet described in published literature, software must be made available to editors and reviewers. We strongly encourage code deposition in a community repository (e.g. GitHub). See the Nature Research guidelines for submitting code & software for further information.

Data

Policy information about availability of data

All manuscripts must include a data availability statement. This statement should provide the following information, where applicable:

- Accession codes, unique identifiers, or web links for publicly available datasets
- A list of figures that have associated raw data
- A description of any restrictions on data availability

All data sources used in this manuscript are publicly available. Links for each dataset are available at the links below. Code for the analysis is available at: [repository published long with the manuscript].
SARS-CoV-2 and Cancer
Rugge M, Zorzi M, Guzzinati S. Malignancy in SARS-CoV2 infection. 2020; published online Oct 26. DOI:10.6084/m9.figshare.12666698.v4. Accessed Nov 23, 2020.

Avian influenza A (H5N1)
Fiebig L, Soyka J, Buda S, Buchholz U, Dehnert M, Haas W. Avian influenza A(H5N1) in humans - line list. Publ. Serv. Robert Koch Inst. 2011; published online Aug 12. DOI:10.25646/7661. Accessed Nov 23, 2020.

Diabetes
UCI Machine Learning. Pima Indians Diabetes Database. Kaggle.com. 2016. https://www.kaggle.com/uciml/pima-indians-diabetes-database (accessed Nov 23, 2020).

Electronic Medical Records
Johnson A, Pollard T, Mark R. MIMIC-III Clinical Database v1.4. PhysioNet. 2016. doi.org/10.13026/C2XW26. Accessed Nov 23, 2020.

Heart Failure
Chicco D. Heart failure clinical records Data Set. UCI Machine Learning Repository. 2020. https://archive.ics.uci.edu/ml/datasets/Heart+failure+clinical+records (accessed Dec 1, 2020)

Bacteraemia
Harris P. Risk factors for relapse or persistence of bacteraemia caused by Enterobacter spp.: a case-control study, https://doi.org/10.7910/DVN/56NCVU, Harvard Dataverse, V1 (2017). Accessed Nov 23, 2020.

Azithromycin in Infants
Lin Y. Replication Data for: MORDOR Infant Adverse Event Survey Data. Harvard Dataverse, V2; doi:10.7910/DVN/MQYM5S. Accessed Nov 23, 2020.

Extrapulmonary Tuberculosis
Ohene S-A. Ghana Extra-pulmonary TB data set.tab. Replication Data Extra-pulmonary Tuberc. a Retrosp. study patients Accra, Ghana. doi:10.7910/DVN/TA1OII/ZSVFGO. (2019). Accessed Nov 23, 2020.

Field-specific reporting

Please select the one below that is the best fit for your research. If you are not sure, read the appropriate sections before making your selection.

☒ Life sciences ☐ Behavioural & social sciences ☐ Ecological, evolutionary & environmental sciences

For a reference copy of the document with all sections, see nature.com/documents/nr-reporting-summary-flat.pdf

Life sciences study design

All studies must disclose on these points even when the disclosure is negative.

Sample size	Following work we are replicating here.
Data exclusions	No exclusions beyond done in the work we are replicating here.
Replication	All findings replicated

Randomization	Following work we are replicating here.
Blinding	Following work we are replicating here.

Reporting for specific materials, systems and methods

We require information from authors about some types of materials, experimental systems and methods used in many studies. Here, indicate whether each material, system or method listed is relevant to your study. If you are not sure if a list item applies to your research, read the appropriate section before selecting a response.

Materials & experimental systems

n/a	Involved in the study	
☒	☐	Antibodies
☒	☐	Eukaryotic cell lines
☒	☐	Palaeontology and archaeology
☒	☐	Animals and other organisms
☐	☒	Human research participants
☐	☒	Clinical data
☒	☐	Dual use research of concern

Methods

n/a	Involved in the study	
☒	☐	ChIP-seq
☒	☐	Flow cytometry
☒	☐	MRI-based neuroimaging

Human research participants

Policy information about studies involving human research participants

Population characteristics	Following work we are replicating here.
Recruitment	Following work we are replicating here.
Ethics oversight	N/A; Following work we are replicating here.

Note that full information on the approval of the study protocol must also be provided in the manuscript.

Clinical data

Policy information about clinical studies

All manuscripts should comply with the ICMJE guidelines for publication of clinical research and a completed CONSORT checklist must be included with all submissions.

Clinical trial registration	Following work we are replicating here.
Study protocol	Following work we are replicating here.
Data collection	Following work we are replicating here.
Outcomes	Following work we are replicating here.
